# 40S ribosome profiling reveals distinct roles for Tma20/Tma22 (MCT-1/DENR) and Tma64 (eIF2D) in 40S subunit recycling

**DOI:** 10.1038/s41467-021-23223-8

**Published:** 2021-05-20

**Authors:** David J. Young, Sezen Meydan, Nicholas R. Guydosh

**Affiliations:** 1 Laboratory of Biochemistry and Genetics, National Institute of Diabetes and Digestive and Kidney Diseases, National Institutes of Health, Bethesda, MD USA; 2 Postdoctoral Research Associate Training Program, National Institute of General Medical Sciences, National Institutes of Health, Bethesda, MD USA

**Keywords:** RNA, Ribosome

## Abstract

The recycling of ribosomes at stop codons for use in further rounds of translation is critical for efficient protein synthesis. Removal of the 60S subunit is catalyzed by the ATPase Rli1 (ABCE1) while removal of the 40S is thought to require Tma64 (eIF2D), Tma20 (MCT-1), and Tma22 (DENR). However, it remains unclear how these Tma proteins cause 40S removal and control reinitiation of downstream translation. Here we used a 40S ribosome footprinting strategy to directly observe intermediate steps of ribosome recycling in cells. Deletion of the genes encoding these Tma proteins resulted in broad accumulation of unrecycled 40S subunits at stop codons, directly establishing their role in 40S recycling. Furthermore, the Tma20/Tma22 heterodimer was responsible for a majority of 40S recycling events while Tma64 played a minor role. Introduction of an autism-associated mutation into *TMA22* resulted in a loss of 40S recycling activity, linking ribosome recycling and neurological disease.

## Abstract (web summary)

Following the termination of translation at stop codons, the eukaryotic 60S subunit of the ribosome is removed by the ATPase ABCE1. Here using 40S ribosome footprinting the authors provide a direct demonstration that the yeast orthologs of eIF2D, MCT-1, and DENR recycle the 40S subunits.

## Introduction

Biogenesis of 40S and 60S ribosomal subunits is an energetically expensive process for the cell^1^. To ensure efficient protein synthesis, each ribosomal subunit is expected to be repeatedly reused during translation. In particular, as the two ribosomal subunits contain 79 proteins, the ribosome must be recycled at least that many times^2^. For each protein synthesis event, 80S ribosomes are first assembled from 40S and 60S subunits at start codons and later disassembled (or recycled) at stop codons after the completed protein is released from the ribosome. While the identity of some of the protein factors that facilitate ribosome recycling are known, their particular mechanism and role in cellular homeostasis is not well understood.

During the assembly, or initiation, phase of translation, the 40S subunit in association with a number of initiation factors and the methionyl initiator tRNA (Met-tRNA_i_^Met^) forms a 43S pre-initiation complex (PIC). The 43S PIC is recruited to the 5′ end of the mRNA via interactions with the m^7^G cap and eIF4F, forming a 48S PIC. It then scans along the 5′ untranslated region (5′UTR) of the mRNA until a start codon is recognized. Next, recruitment of the 60S subunit to the PIC is facilitated by the universally conserved GTPase eIF5B (a homolog of bacterial IF2)^3^. The assembled 80S ribosome then synthesizes the protein encoded by the open reading frame (ORF).

Following completion of protein synthesis, the post-termination ribosome must undergo a recycling process that is the functional inverse of initiation. The first stage of recycling, splitting of the 80S ribosome into a free 60S subunit and a tRNA/mRNA bound 40S subunit, is catalyzed by ABCE1 (Rli1 in yeast)^4–6^ and further enhanced by eIF3j (Hcr1 in yeast)^7^. The second stage, dissociation of the deacylated tRNA and mRNA from the 40S subunit, has been reconstituted in vitro with eIF2D (also referred to as ligatin) alone, or a heterodimer composed of MCT-1 (also referred to as MCTS1) and DENR, which are homologous to the N- and C-termini of eIF2D, respectively^8^. However, it remains unknown whether eIF2D and the MCT-1/DENR heterodimer have overlapping or independent roles in translation^9^.

We previously found that deletion of the yeast genes that encode Tma64, Tma20, and Tma22 (orthologs of eIF2D, MCT-1, and DENR, respectively) resulted in phenotypes that were consistent with a ribosome recycling defect in vivo^10^, such as queuing of elongating 80S ribosomes upstream of the stop codon and reinitiation of translation in 3′UTRs. Based on this indirect evidence, we hypothesized that 80S ribosomes formed queues behind an unrecycled 40S subunit or an 80S ribosome that arrested at the stop codon but fell apart in our sample preparation. However, we were unable to directly show whether the obstacle was derived from the post-termination ribosome or some other RNA-binding protein. In addition, we found reinitiation in 3′UTRs required a downstream AUG codon in some, but not all, cases and therefore raised the question of whether the reinitiation process takes place via a canonical AUG-dependent 40S scanning process or relied on an alternative, such as 80S scanning, that has been observed in cells depleted of 60S recycling factors^6,7^. The mechanism by which the Tma proteins regulate 40S recycling therefore remains ambiguous.

Apart from this potential role in recycling, eIF2D and MCT-1/DENR have also been proposed to carry out other roles in translation. Sequence homology between eIF1, DENR, and the C-terminus of eIF2D, as well as similarity in the conformations adopted when bound to the 40S subunit, suggest a possible role in initiation^11,12^. In support of this idea, these proteins were shown in vitro to promote recruitment of Met-tRNA_i_^Met^ to specialized mRNA/40S complexes in the absence of the canonical eIF2·GTP·Met-tRNA_i_^Met^ ternary complex^8,13^. These factors have therefore also been proposed to offer an alternate mechanism to promote initiation under conditions where functional eIF2 levels were reduced. DENR and MCT-1 have been shown to promote translation reinitiation at ORFs that are preceded by short upstream open reading frames (uORFs) with strong Kozak sequences^14,15^. In particular, DENR was shown to be required for reinitiation after uORFs on genes that regulate circadian rhythms and for maintenance of the correct circadian period^16,17^. These alternative roles for eIF2D and MCT-1/DENR seem to be at odds with these factors playing a role in ribosome recycling. However, it may be possible to harmonize these divergent findings if these proteins are able to remove tRNA but leave the 40S bound to mRNA in some cases. In this scenario, deletion or depletion of these factors would prevent the unrecycled 40S ribosome from acquiring Met-tRNA_i_^Met^ and reinitiating at a main ORF start codon. Whether the Tma proteins play a role primarily in initiation or recycling, or are perhaps critical in both processes, remains an open question.

As is the case with many translation factors, MCT-1, DENR, and eIF2D have all been proposed to be oncogenes^18–21^. In addition, mutations in DENR (C37Y and P121L) have been identified in patients with autism spectrum disorders (ASD)^22,23^. The C37Y mutation eliminates one of the highly conserved cysteine residues that forms the zinc-binding domain of DENR that physically interacts with MCT-1 and the P121L mutation lies within the β1-loop that extends near the codon-anticodon interaction region^22–24^. Depletion of DENR using short-hairpin RNAs resulted in disruption of mouse cortical neuron migration, differentiation, and dendritic spine density. These neuronal defects could not be rescued by expression of DENR carrying the C37Y mutation. Expression of DENR-P121L resulted in partial rescue of the defects^23^. Whether these mutations result in defects in 40S recycling or other stages of translation remains unknown.

Here we used a 40S ribosome profiling approach similar to TCP-seq^25^ and other recently published 40S profiling strategies^26–28^ to directly observe key intermediates in 40S ribosome recycling. 40S profiling of strains lacking the Tma factors (Tma64, Tma20, and Tma22) revealed substantial accumulation of 40S subunits at stop codons, establishing their role as bona fide 40S subunit recycling factors in vivo. During revision of this manuscript, complementary results from 80S and 40S ribosome profiling were published for DENR, the mammalian ortholog of Tma22^29^. Further analysis of individual knockout strains revealed a bias in recycling workload, with Tma20/Tma22 being responsible for the majority of the activity. Introduction of the autism-associated mutation C37Y into yeast Tma22 resulted in a loss of recycling activity, thus suggesting a role for ribosome recycling in ASD.

## Results

### 40S ribosome profiling allows quantification of 40S ribosomal subunits in vivo

To study the complementary steps required for ribosomes and mRNA to associate during translation initiation and dissociate during recycling, we have utilized a 40S ribosome profiling method (Supplementary Fig. 1a, see also Methods) based on 80S ribosome profiling^30,31^ and an earlier 40S profiling approach, TCP-seq^25^. Standard 80S ribosome profiling uses high-throughput sequencing of ribosome-protected mRNA fragments (referred to as ribosome footprints) to assess gene expression and the mechanism of translation. Profiling of 40S subunit-protected mRNA fragments is complicated by the fact that 40S-mRNA complexes are intrinsically less stable than 80S ribosomes and can be mechanically dissociated during ultracentrifugation. This difficulty can be overcome by cross-linking cells with 1% formaldehyde (CH_2_O)^32^ as was used to stabilize ribosomal complexes in TCP-seq^25^ and a number of updated versions of this method that have recently been published^26–29^.

In our approach (Supplementary Fig. 1a), we cross-linked log-phase yeast cells with 1% formaldehyde for 1 h and lysed them in a cryo-mill. Lysates were digested with RNase I and separated by sucrose gradient centrifugation. Footprints measuring 15–80 nt were selected, based on previous studies of digested ribosomal complexes^33,34^. Sequencing libraries were then constructed with the same protocol used for standard 80S footprinting, as described previously^31^. This approach allows unambiguous identification of the 3′ end of the footprint by ligating it to a known linker sequence and therefore improves on the original approach that relied on polyadenylation^25^. These linkers also contain unique molecular identifiers (UMIs) that allow removal of bias caused by PCR duplicates. We eliminated rRNA contamination with the Illumina Ribo-Zero Gold rRNA Removal Kit. We found the percentage of reads that did not align to ncRNAs ranged from 5.6 to 14.5% (Supplementary Table 1) compared to 1.54% and 2.06% for the original implementation of TCP-seq^25^, demonstrating that hybridization approaches can help reduce contaminating rRNA. Most remaining contaminants consisted of tRNA, suggesting further improvements might be achievable by including oligonucleotides complementary to these tRNA sequences in the depletion mix.

### Knockout of *RPL11B* enhances 40S peaks on start codons

**Fig. 1:**
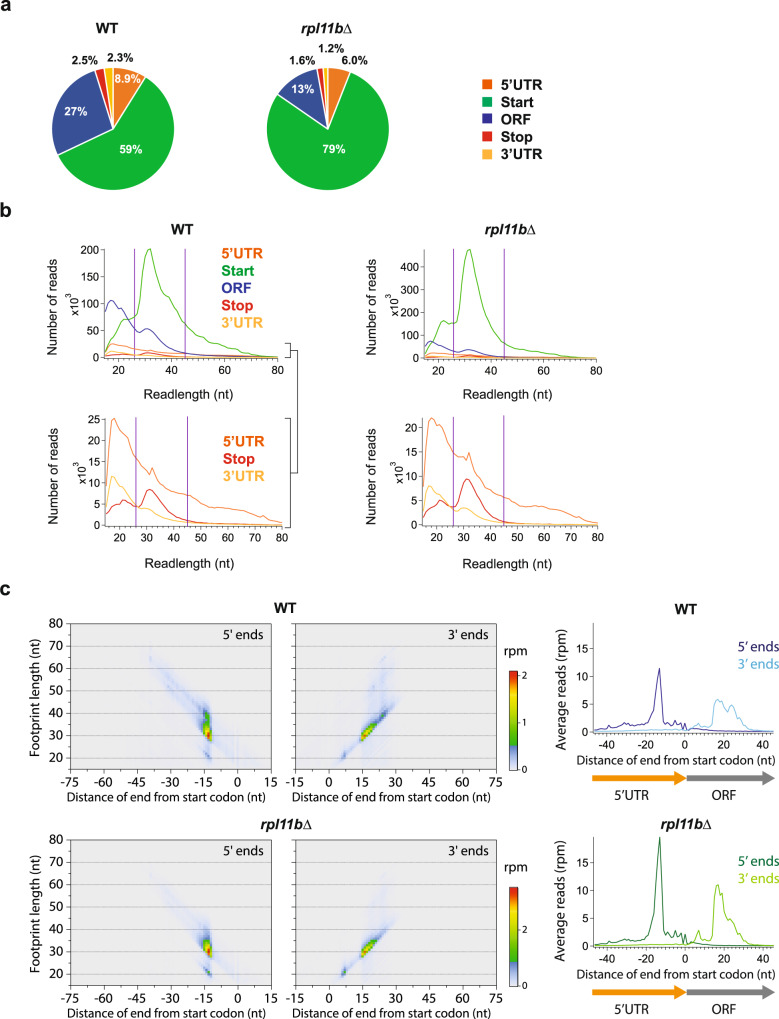
Quantification of 40S ribosomal footprints across the transcriptome. **a** Proportion of 40S footprints that mapped to different mRNA regions from WT and *rpl11b*∆ strains. See Methods for definitions of regions. **b** Histograms of footprint lengths that were mapped to each of the different mRNA regions (upper plots). The lower plots show a magnified view for the 5′UTR, Stop, and 3′UTR regions. The purple lines encompass the range of footprint lengths used for most analysis (26–45 nt). **c** Two-dimensional metagene plots show the correlation between footprint length and mapping position of 5′ end aligned (left panels) and 3′ end aligned (middle panels) footprints. These plots include data from all genes aligned by their start codons. Differences in footprint length are due to 3′ end variability (mapping position mostly does not vary by length in left panels, in contrast to center panels). Data for WT (top) and *rpl11b*∆ (bottom) are shown. Loss of *RPL11B* enhances main peak at the start codon, but diagonal tail corresponding to reads extended on their 5′ ends becomes fainter (left panels of 5′-aligned reads). Corresponding one-dimensional metagene plots are shown (right panels) at start codons. These plots combine the length information (for all lengths, 15–80 nt) into a single trace. nt nucleotide, rpm reads per million. Source data are provided as a Source Data file. See also Supplementary Fig. 1.

We initially tested our 40S ribosome profiling approach on WT and *rpl11b* deletion strains of yeast in duplicate (two biological replicates). Deletion of *RPL11B*, one of two genes that encode the large ribosomal subunit protein Rpl11p in *S. cerevisiae* (uL5), results in reduced levels of 60S subunits. This shortage of 60S subunits extends the time a 48S PIC must wait for a 60S subunit to join with it at a start codon, consistent with the presence of half-mer peaks in polysome profiles^35^ that signal the accumulation of 48S PICs at mRNA start codons^36^. We expected this defect to manifest as an increase in 40S footprint occupancy at start codons in the knockout data and therefore serve as a control for defining 40S peaks in our method. To test this prediction, we computed the fraction of footprints that mapped to annotated transcript features (defined in the Methods section). The majority of footprints in WT cells mapped to 5′UTRs (8.9%) and start codons (59%) and to a much lesser extent to stop codons (2.5%) and 3′UTRs (2.3%) (Fig. 1a). The *rpl11b*∆ strain showed an increase in the relative number of 40S footprints mapping to the start codon (59% in WT to 79% in *rpl11b*∆) (Fig. 1a), consistent with the expected decrease in the pool of free 60S subunits^35,36^. A percentage of 40S footprint reads mapped to ORFs, as has been previously observed and suggested to result from residual dissociation of 80S ribosomes during sedimentation^25^.

We next evaluated mRNA footprint lengths protected by 40S subunits. The majority of 40S footprints were similar in length to 80S ribosome footprints (~32 nt vs ~28 nt) (Fig. 1b, top panels). A small peak of ~21 nt 40S footprints was observed, particularly in ORFs. As 80S ribosomes undergoing elongation are known to protect 21-nt footprints when the A site is open^37^, it is conceivable that these footprints originate from 80S ribosomes that fell apart during sedimentation. We also noted reads <20 nt in some samples that included contamination from fragmented tRNA (see Methods). In addition, the distribution of footprint lengths mapping to start codons (Fig. 1b, top panels) and 5′UTRs (Fig. 1b, bottom panels) included a tail of longer lengths, suggesting that 40S complexes undergoing initiation can protect longer footprints, perhaps due to the presence of bound initiation factors. These extended 40S footprints are consistent with previous reports of 48S complexes protecting longer lengths of RNA than 80S ribosomes^38–40^.

We then mapped reads to the genome and to splice junctions and averaged the read counts across genes that had been aligned by their start codons. These averages were used to create average or metagene plots (Fig. 1c). We performed this analysis for 5′ and 3′ ends for each footprint length independently to reveal which end of the footprint was responsible for variations in overall length (Fig. 1c, left/center panels). We found the variation mainly occurred on the 3′ end at start codons (data mainly fall along a diagonal when data are aligned by 3′ ends, in contrast to data plotted by 5′ ends), consistent with what was observed for TCP-seq^25^. This 3′ end variability contrasts with conventional 80S ribosome profiling in yeast, where 5′ end variability is greater^41^. However, we did note a minor population of very long (>45 nt) footprints that primarily varied at the 5′ end around start codons (Fig. 1c left, faint diagonal tail in 5′-aligned plot). Results of metagene analysis of the *rpl11b*∆ data (Fig. 1c left) were similar except the peak at start codons was stronger for footprints of ~32 nt (note the change in color scale) but not in the population of longer footprints (5′ aligned panels, diagonal tail in the WT is relatively fainter in *rpl11b*∆). This observation is consistent with the overall enrichment of footprints around start codons in *rpl11b*∆ (Fig. 1a) due to the shortage of mature 60S subunits in the cell. It also suggests that the very long (>45 nt) footprints are not derived from the population of 40S subunits that are waiting at the start codon for a 60S subunit. Instead, they may be derived from a PIC at an earlier stage of initiation that is kinetically separate from a later stage that is made more abundant by slow 60S joining. We also performed aggregate metagene analysis with these data across all read lengths (15–80 nt; Fig. 1c, right). This analysis also revealed generally greater variability on 3′ ends in the form of broader peaks at start codons. A zoomed version of this plot confirmed that reads mapping to the ORF likely came from 80 S ribosomes that dissociated, due to the clear signature of 3-nt periodicity (Supplementary Fig. 1b).

These results confirm that our 40S ribosome profiling method is capable of detecting and quantifying changes in 40S subunit distribution. For consistency, 5′ end alignment was used throughout the study for both 40S and 80S profiling data. For enhanced precision, we also limited further analysis (unless noted otherwise) to reads measuring 26–45 nt (denoted by purple lines, Fig. 1b).

### 40S peaks mark start codons and N-terminal protein extensions

**Fig. 2:**
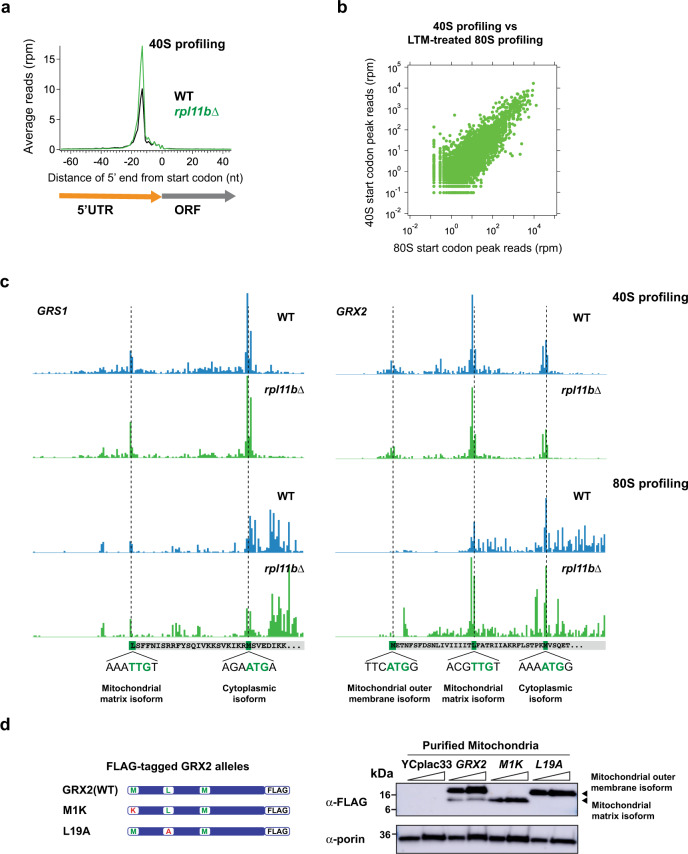
40S PICs are enriched on start codons due to loss of *RPL11B* and predict downstream translation. **a** Average ribosome footprint occupancy at start codons increased (~2×) for 40S data for cells lacking *RPL11B*. Footprints of 26–45 nt plotted by 5′ ends. **b** Comparison of 40S start codon peak levels (total footprints at the start codon) with LTM-treated 80S start codon peak levels (total footprints at the start codon) from Eisenberg et al. (2020)^42^ revealed a strong correlation (Pearson *R*^2^ = 0.61) showing that 40S peaks can be used to identify translation start sites. Each point represents the data for one gene. Footprint reads were quantitated after shifting 40S data by 14 nt and 80S data by 13 nt. **c** Data from 40S and 80S profiling data corresponding to the *GRS1* and *GRX2* genes. The 40S footprint occupancy peaks at cognate AUG and near-cognate UUG codons reveal the N-terminal extensions that are apparent in the 80S data. Gene annotations (DNA sequences and amino acids encoded by cognate tRNAs) are drawn to correspond to ribosome P sites. **d** Analysis of mitochondria that were purified from BY4741 cells expressing FLAG-tagged versions of either WT *GRX2*, *GRX2-M1K*, or *GRX2-L19A* from the sc expression vector YCplac33. Diagrams of the FLAG-tagged *GRX2* alleles showing the three potential translation start sites and their mutations are shown on the left. TCA protein extracts from the purified mitochondria were subjected to western analysis using mouse monoclonal antibodies against FLAG (top blot) or VDAC1/Porin (mitochondrial control, bottom blot). Two amounts of extracts (1× and 2×) were loaded in each lane pair. The M1K mutation results in loss of expression of the mitochondrial outer membrane Grx2 isoform and the L19A mutation results in loss of expression of the mitochondrial matrix Grx2 isoform. Westerns were repeated twice on two independently grown cultures for each condition to ensure replicability. nt nucleotide, rpm reads per million, kDa kilodalton. Source data are provided as a Source Data file. See also Supplementary Fig. 2.

We next investigated whether our method could be used as a tool for confirming existing annotations of start codons and also predicting novel translation start sites. One advantage of 40S ribosome profiling over 80S profiling is that the majority of 40S reads occur at start codons whereas 80S profiling reads also come from elongating ribosomes found in ORFs. Internal start codon peaks (marking short internal ORFs or N-terminal truncations) tend to get obscured in the 80S data by neighboring reads from elongating ribosomes. The general absence of 40S footprints within ORF regions overcomes this problem and, combined with the enrichment of 48S PICs at start codons in the *rpl11b*∆ strain, suggests that peaks in 40S profiling data could be a useful marker for initiation sites. To test this, we again performed metagene analysis at start codons for WT and *rpl11b*∆ datasets, now using the focused 26–45 nt footprint lengths discussed above (Fig. 2a) and overlaid the data for comparison. Similar to the previous analysis of the entire footprint range (Fig. 1c), the 40S peak is enhanced by approximately twofold by the loss of *RPL11B* and the overlay directly illustrates the utility of this approach of deleting *RPL11B* to enhance 40S peaks at start codons.

To test the utility of our approach for identifying translation start sites we compared our 40S profiling method to another recently published method of identifying start sites based on 80S profiling of lactimidomycin (LTM)-treated cells^42^. LTM preferentially inhibits post-initiation 80 S ribosomes while allowing elongating 80S ribosomes to run off, resulting in enrichment of 80S ribosome footprints at translation start sites^43,44^. We calculated the number of reads in peaks on canonical start codons for each gene for both our 40S profiling data and the LTM-treated 80S profiling data and found a strong correlation between the two datasets (Fig. 2b; Pearson *R*^2^ = 0.61). This is a high level of correlation given the different strain backgrounds used in the two studies (BY4741 in the current study and SK1 in Eisenberg et al. 2020^42^) and the difference in ploidy between the strains (BY4741 is haploid and SK1 is diploid). This result shows that 40S profiling compares favorably to other global methods of experimentally determining translation start sites.

We also tested whether our data could predict known cases where multiple start sites are used to encode protein isoforms with N-terminal extensions. We found several cases where this was true. For example, two major 40S occupancy peaks were observed at alternative start codons within the *GRS1* transcript, which encodes both the cytoplasmic and mitochondrial isoforms of glycyl-tRNA synthetase. The N-terminally extended protein isoform contains sequences required for targeting Grs1 to the mitochondria (Fig. 2c, left)^45^. Similar peaks were also observed for *ALA1*, encoding the mitochondrial and cytoplasmic isoforms of alanyl-tRNA synthetase (Supplementary Fig. 2a)^46^. The *CCA1* gene encodes the mitochondrial, nuclear, and cytoplasmic isoforms of tRNA nucleotidyltransferase^47,48^. We were able to observe 40S occupancy peaks at all three start codons in the N-terminus of the *CCA1* gene (Supplementary Fig. 2b). In some cases, the corresponding 80S data also show some evidence of translation. However, as discussed above, the signal is poorer in comparison and therefore highlights the power of 40S footprinting, particularly in the *rpl11b*∆ strain, for finding these rare translation initiation events.

We also found evidence for a novel start site that can account for the unusual localization pattern of the glutaredoxin protein, which is encoded by the *GRX2* gene. The gene is known to have two translation start sites but protein isoforms are known to be localized to three different cellular compartments: the cytoplasm, mitochondrial matrix, and the mitochondrial outer membrane^49^. It has previously been suggested that the first AUG generates an isoform with a long N-terminal extension that includes a hydrophobic region of unknown function followed by a motif for localization to the mitochondrial matrix. The authors proposed that the hydrophobic region interfered with mitochondrial import and could trap some of the protein in the mitochondrial outer membrane. Any protein that was not trapped was proposed to be imported into the mitochondrial matrix. In addition, a short isoform was proposed to be initiated from the second AUG and localized to the cytoplasm due to the absence of the localization sequence^49^. In contrast to this model, our 40S ribosome profiling suggests that the protein localized to the mitochondrial matrix may not rely on inefficient import, but instead is synthesized as an isoform of intermediate length that lacks the hydrophobic region but retains the mitochondrial localization signal. We observed a translation initiation site at a UUG start codon, in between the two AUG codons, that would produce this isoform (Fig. 2c, right). We propose that the hydrophobic region may instead be a transmembrane helix that efficiently targets the long isoform of the protein to the mitochondrial outer membrane.

To test this hypothesis we cloned the *GRX2* gene into a single-copy yeast expression plasmid YCplac33^50^. We then used site-directed mutagenesis to insert the DNA sequence for a FLAG-tag at the C-terminus of the protein to allow the detection of the various Grx2 isoforms by immunoblotting with an anti-FLAG antibody. Site-directed mutagenesis was used to separately mutate the methionine codon that we hypothesized initiates translation of the mitochondrial outer membrane Grx2 isoform to a lysine codon (M1K; ATG → AAA), and the near-cognate leucine codon that we hypothesized initiates the mitochondrial matrix Grx2 isoform to an alanine codon (L19A; TTG → GCT) (Fig. 2d left). We then transformed these plasmids into the BY4741 strain and purified mitochondria from the transformed cells. Western blotting of the mitochondrial protein extract from the strain transformed with the WT *GRX2*-FLAG allele revealed two bands: a larger band corresponding to the mitochondrial outer membrane Grx2 isoform, and a smaller band corresponding to the mitochondrial matrix Grx2 isoform (Fig. 2d right, lanes 3–4). In agreement with these assignments, the M1K mutation resulted in loss of the larger band corresponding to the mitochondrial outer membrane Grx2 isoform (Fig. 2d right, lanes 5–6), and the L19A mutation resulted in loss of the smaller band corresponding to the mitochondrial matrix isoform (Fig. 2d right, lanes 7–8). These results confirm that the novel UUG translation start site predicted by 40S ribosome profiling is responsible for production of the mitochondrial matrix Grx2 isoform.

Overall, these examples show that 40S profiling can be used as a tool to map previously unidentified N-terminal protein extensions that are not readily apparent in 80S profiling data.

### 40S peaks reveal initiation at upstream ORFs (uORFs)

**Fig. 3:**
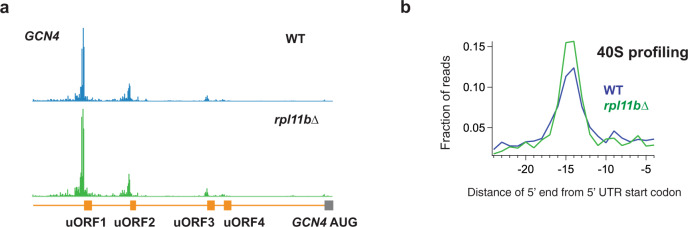
40S reads are enriched on start codons at upstream ORFs (uORFs). **a** 40S ribosome footprint profiles of the 5′UTR of the *GCN4* gene showing 40S footprint occupancy peaks at 5′UTR uORF AUG codons. Gene annotations are drawn to correspond to data for ribosome P sites. **b** Average fraction of 40S ribosome occupancy in a window surrounding 5′UTR AUG codons. Data shown for WT and *rpl11b*∆ cells. Source data are provided as a Source Data file. See also Supplementary Fig. 3.

We next analyzed 40S peaks at uORF start codons. In particular, we found 40S occupancy at the four known uORF AUG codons in the 5’UTR of the *GCN4* gene (Fig. 3a)^51^. Interestingly, a population of 5′ extended footprints (>45 nt) was observed at the *GCN4* uORF1 AUG codon (Supplementary Fig. 3a-b). The sequences upstream of uORF1 (and uORF2) contain reinitiation promoting elements (RPEs) that have been shown to be important for reinitiation after termination (reviewed in Gunisova et al. 2018^52^). These RPEs form secondary structures that bind to the 40S subunit. Binding by some of the RPEs may protect the reinitiation elements from RNase I digestion, thus producing the observed extended ribosome footprints. More broadly, averaging of 40S reads across all 5′UTR AUG codons revealed an apparent 40S occupancy peak in both WT and *rpl11b*∆ datasets (Fig. 3b), further confirming that our 40S ribosome profiling method can detect initiation events at 5′UTR uORFs.

### The Tma factors recycle 40S subunits at stop codons in vivo

Our previous study used 80S ribosome profiling to indirectly suggest that the Tma factors were required in some way for the recycling of ribosomes at stop codons^10^. We found that in either a *tma64*∆*tma20*∆ or *tma64*∆*tma22*∆ strain, translation could reinitiate in 3′UTRs and that 80S ribosomes queued upstream of stop codons. These double deletion strains (*tma*∆∆) were used to control for any redundancy between Tma64 and Tma20 or Tma22. To directly assess how the Tma proteins are involved in recycling of the 40S subunit, we used 40S ribosome profiling to quantify the level of 40S subunits stalled at stop codons in the *tma*∆∆ strains in two biological replicates.

**Fig. 4:**
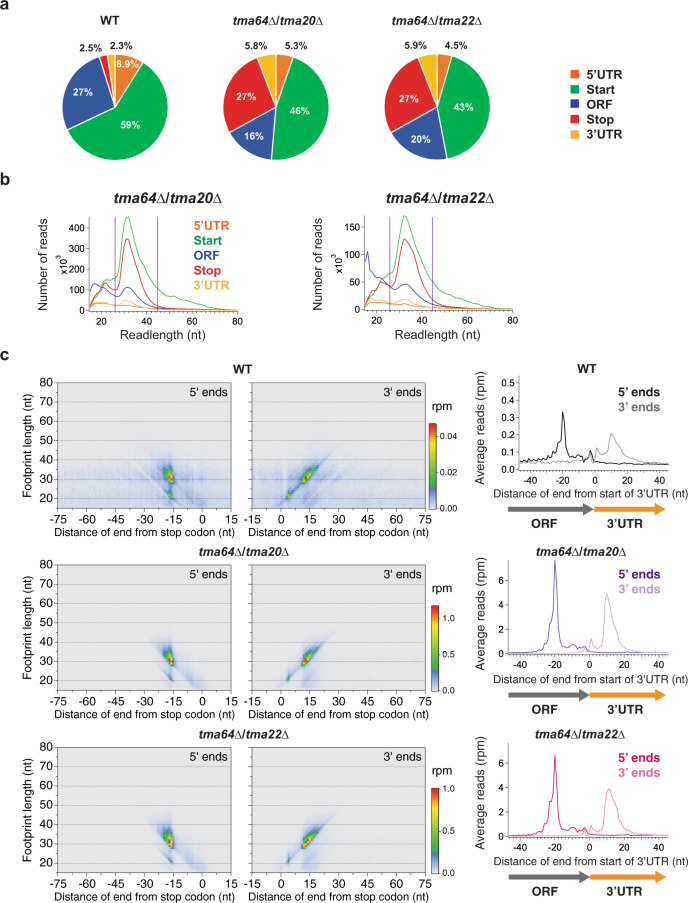
Deletion of *TMA64*, *TMA20*, and *TMA22* results in accumulation of 40S footprints at stop codons. **a** Proportion of 40S footprints that mapped to different mRNA regions from *tma64*∆/*tma20*∆ and *tma64*∆/*tma22*∆ strains, and for reference, the WT strain. **b** Histograms of footprint lengths that were mapped to each of the different mRNA regions (upper plots). The purple lines encompass the range of footprint lengths used for most analysis (26–45 nt). **c** Two-dimensional metagene plots show the correlation between footprint length and mapping position of 5′ end aligned (left panels) and 3′ end aligned (middle panels) footprints. These plots include data from all genes aligned by their stop codons. Differences in footprint length are due to 3′ end variability (mapping position mostly does not vary by length in left panels, in contrast to center panels). Data for WT (top) and *tma*∆∆ strains are shown (middle and bottom). Corresponding one-dimensional metagene plots are shown (right panels) at stop codons. These plots combine the length information (for all lengths, 15–80 nt) into a single trace. nt nucleotide, rpm reads per million. Source data are provided as a Source Data file.

Our data revealed a dramatic increase in the fraction of 40S footprints mapping to stop codons relative to WT (2.5% in WT to 27% in the *tma*∆∆ strains; Fig. 4a) and an increase in 40S subunits in the 3′ UTR (2.3% in WT to ~6% in the *tma*∆∆ strains; Fig. 4a). These twin findings support a model where unrecycled 40S subunits accumulate at stop codons in the knockout strains and reinitiate translation at short ORFs in the 3′UTR. The major footprint length of reads that map to stop codons (~32 nt) was similar to those mapping to start codons (Fig. 4b). However, the distribution around stop codons lacked the tail of longer lengths observed for the distribution around start codons. This may be due to the different complement of proteins required for translation initiation vs ribosome recycling. We also found that the variation in 40S footprint length appeared to mainly derive from variability at 3′ ends, as we previously found for footprints around start codons (Fig. 4c).

**Fig. 5:**
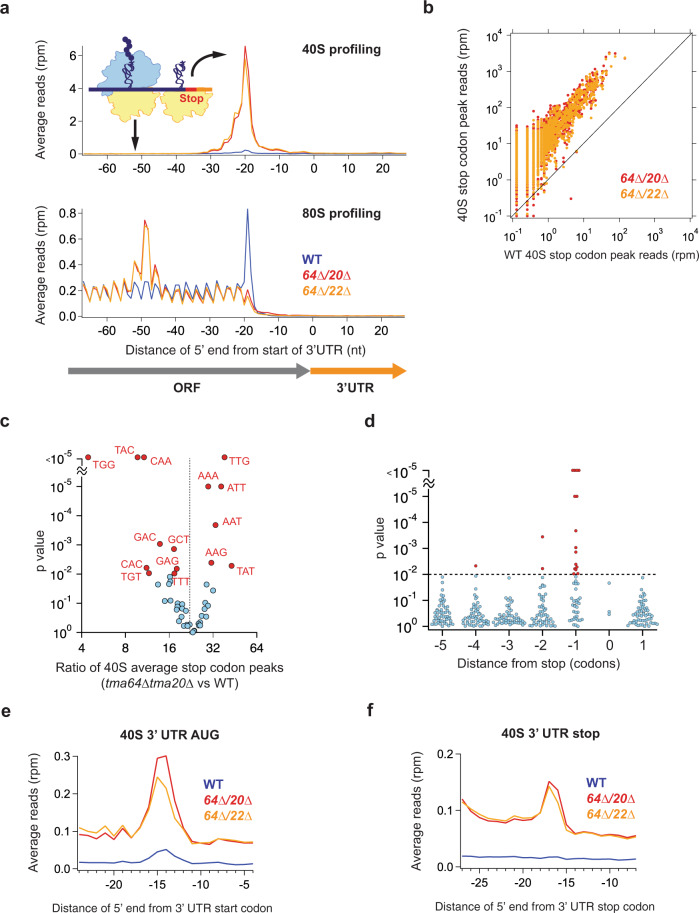
The Tma proteins recycle 40S subunits at stop codons in vivo. **a** Average 40S (top) and 80S (bottom) ribosome footprint occupancy from all genes aligned by their stop codons reveals increased 40S footprints on stop codons and 80S footprints positioned further upstream in the *tma*∆∆ strains. Footprints plotted by 5′ ends. 80S data have been previously published^10^. The schematic shows a model of the recycling defect in the *tma*∆∆ strains where a 40S subunit is stalled at the stop codon and an 80S ribosome is queued behind it. **b** Correlation analysis of 40S stop codon peak heights (total count of footprints at the stop codon) between WT strain and *tma*∆∆ strains. The plot reveals that the peaks on stop codons are broadly higher across the transcriptome in the *tma*∆∆ strains (data points shifted above the diagonal). Each point represents the data for one gene. Footprint reads were quantitated by shifting them by 14 nt and then counting reads around the stop codon. **c** Average ratio of 40S stop codon peaks (*tma64*∆*tma20*∆ vs WT) are plotted against their significance (*p* value) as determined by bootstrapping (see Methods) for penultimate codons with at least ten occurrences. Significant codons (DNA sequences) are indicated (red) at the 99th confidence interval. Dotted line indicates average increase across the entire transcriptome of 23.4-fold. **d** Average ratio of 40S stop codon peaks (*tma64*∆*tma20*∆ vs WT) were computed for subsets of particular codons near the end of the ORF. Significant codons are indicated (red) at the 99th confidence interval (dotted line). The penultimate codon (−1) position has far more codons that significantly change between *tma64*∆*tma20*∆ and WT than other positions. **e** Average 40S ribosome occupancy in a window surrounding 3′UTR AUG codons. **f** Average 40S ribosome occupancy in a window surrounding 3′UTR stop codons. Note that loss of the Tma proteins increases this peak, showing their activity in recycling 40S subunits in the 3′UTR. nt nucleotide, rpm reads per million. Source data are provided as a Source Data file. See also Supplementary Fig. 4.

Consistent with these observations, we saw a sharp increase in the height of the peak of average 40S footprints on stop codons in the metagene plots for both *tma*∆∆ strains (Fig. 5a, top). When 80S profiling data that we previously reported^10^ are averaged in the same way (Fig. 5a, bottom), we again noted an 80S ribosome upstream of the stop codon. Our new 40S profiling data now allows us to complete our model by directly showing how an unrecycled 40S subunit at the stop codon is the block that causes upstream 80S ribosomes to queue (Fig. 5a, schematic). We note that the peak corresponding to the queued 80S ribosome at position −49 nt (measured from the start of the 3′UTR) includes two minor satellite peaks at −46 and −52 nt. These satellites suggest variable packing exists between the 80S and stalled 40S subunit, similar to what has been observed for packing between 80S ribosomes in disomes^53^.

We further examined the distribution and magnitude of the 40S accumulation on stop codons by calculating 40S stop codon peak heights (defined as the total count of footprints at the stop codon, see Methods) for each gene in WT and *tma*∆∆ strains. We found that 40S accumulation on stop codons occurred broadly in both mutant strains and generally in proportion to the size of the existing peak in WT cells (Fig. 5b and Supplementary Fig. 4a-b for replicates). This correlation suggests that loss of these factors slows the step that limits the rate of 40S recycling in WT cells and that the Tma proteins are critical for recycling on nearly all genes. We also checked to see if the increases in stop codon peaks varied between the *tma64*∆*tma20*∆ and *tma64*∆*tma22*∆ strains. We found the peaks were highly correlated, implying little specificity for the loss of *TMA20* vs *TMA22* (Supplementary Fig. 4c).

While stop codon peaks broadly increased for all genes in the *tma*∆∆ strains, the existence of some spread in the data (Fig. 5b, correlation is less than perfect) suggests that other variables may modulate the magnitude of the increase in the *tma*∆∆ strains. A well-known factor that affects translation termination efficiency is the identity of the penultimate codon (codon before the stop codon), potentially due to variability in interactions between the cognate tRNA and the terminating ribosome complex. Specifically, the last two C-terminal amino acids are overrepresented by corresponding tRNAs with hyper-modified bases at positions 32, 34 and 37, locations in the anticodon stem–loop (ASL)^54^. Such interactions could also potentially affect the efficiency of 40S recycling. We therefore investigated the influence of the identity of the penultimate codon on stop codon peaks in the *tma64*∆*tma20*∆ strain. We calculated the average ratio of 40S stop codon peaks (*tma64*∆*tma20*∆ vs WT) for penultimate codons with at least ten occurrences and plotted them against the probability (*p* value determined by bootstrapping, see Methods) that the average is not different from the mean increase for the entire transcriptome (Fig. 5c, dotted line indicates average increase of 23.4-fold). The analysis revealed some penultimate codons significantly increased (right side of the dotted line) or decreased (left side of the dotted line) the stop codon peak ratio (indicated in red; 99^th^ confidence interval) and therefore shows that the penultimate codon can modulate a gene’s dependence on the Tma factors for ribosome recycling. Further analysis showed this dependence was very similar for both *tma*∆∆ mutants (Supplementary Fig. 4d). The penultimate codons that show a strong dependence on 40S recycling factors include several AAN codons. This effect could be driven by slow dissociation of the tRNAs that correspond to these codons, perhaps promoted by stabilizing interactions due to hypermodified positions in the ASL^55^. In contrast, we noted that the TGT codon was among the least dependent on the Tma factors, consistent with previous work suggesting the tRNA^Cys^ is weakly associated with the post-termination 40S complex and may readily dissociate after subunit separation (reviewed in Gunisova et al. 2018^52^).

To determine if any other codon positions around the stop codon affect 40S stop codon peak height, we computed the average ratio of 40S stop codon peaks (*tma64*∆*tma20*∆ vs WT) for subsets of particular codons near the end of the ORF (Fig. 5d). Significant codons are indicated in red at the 99th confidence interval. The penultimate codon (−1) position has far more codons that change between *tma64*∆*tma20*∆ and WT than other positions, consistent with the idea that interactions with the residual P-site tRNA could modulate the dependence on 40S recycling factors. These findings are consistent with recently published results from HeLa cells, which showed that both uORF and main ORF dependence of 40S recycling on DENR is affected by the identity of the tRNA bound to the penultimate codon^29^.

We also explored how loss of the Tma proteins affects reinitiation of translation in 3′UTRs. We previously showed that loss of these proteins leads to increased 80S ribosome footprints in the 3′UTR due to some form of reinitiation^10^. In particular, averaging 80S ribosome profiling data around all 3′UTR AUG codons revealed a density peak in the *tma*∆∆ strains (analysis reproduced in Supplementary Fig. 4e), implying AUG codons are required to support reinitiation in at least some cases. However, it was not clear whether reinitiation was occurring due to reinitiation of scanning by a 40S subunit or 80S ribosome. We therefore averaged 40S data from our *tma*∆∆ strains at 3′UTR AUG codons (Fig. 5e). We noted an increase in these peaks in the *tma*∆∆ strains, directly showing that unrecycled 40S subunits can start scanning in 3′UTR regions and reinitiate at AUG codons.

Furthermore, we were able to detect 40S peaks at 3′UTR stop codons in averaged *tma*∆∆ 40S data (Fig. 4f). This result shows that the Tma factors are also required for recycling after translation of short ORFs downstream of the stop codon (dORFs). The role of the Tma proteins is therefore broadly important for efficient recycling at stop codons.

### Tma20 and Tma22 are more critical for recycling than Tma64

**Fig. 6:**
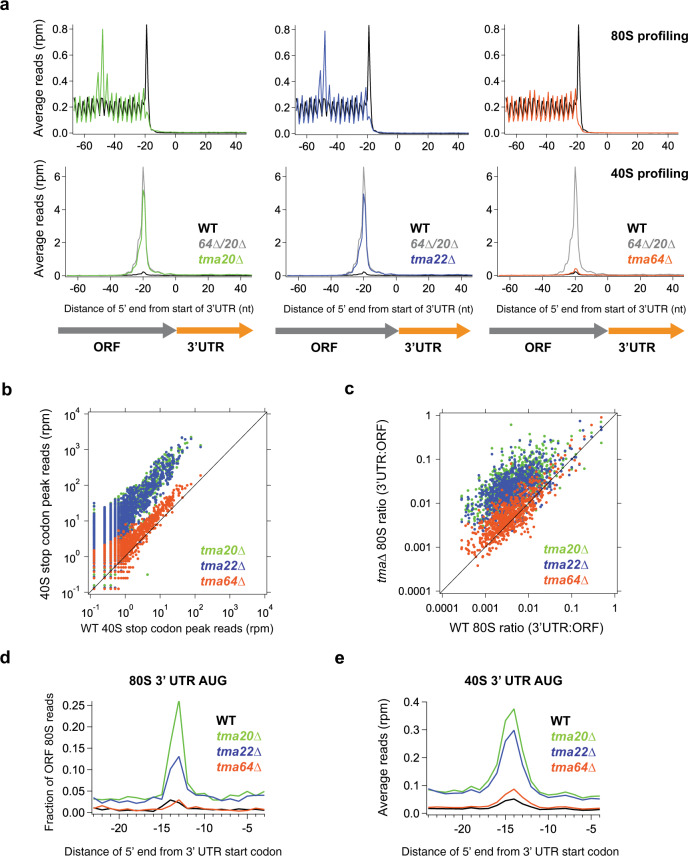
Tma20 and Tma22 are responsible for the majority of 40S recycling at stop codons. **a** Average 80S (top) and 40S (bottom) ribosome footprint occupancy across all genes aligned at their stop codons for the *tma20*∆ (left), *tma22*∆ (middle), and *tma64*∆ (right) strains. The data reveal a queued 80S peak one footprint length behind the stop codon (top) and increased 40S peak on the stop codon (bottom) in the *tma20*∆ and *tma22*∆ strains. These trends are much smaller in the *tma64*∆ strain. Footprints plotted by 5′ ends. **b** Analysis of 40S stop codon peak heights (total count of footprints at the stop codon) showing a strong increase in stop codon peak height in the *tma20*∆ and *tma22*∆ strains (dots above the diagonal) but only a small shift in the *tma64*∆ strain, compared to WT. The lack of outliers indicates the step in 40S recycling slowed by loss of the Tma proteins is also slow in WT cells. Each point represents the data for one gene. Footprint reads were quantitated by shifting them by 14 nt and then counting reads around the stop codon. **c** Ratio of 80S footprint density in 3′UTRs to their respective ORFs is plotted for the individual *tma* deletion vs WT cells. Each point represents the data for one gene. The data are consistent with increased 3′UTR 80S ribosome occupancy for most genes in the *tma20*∆ and *tma22*∆ strains (most dots are above the diagonal) but only a small shift in the *tma64*∆ strain, compared to WT. **d** Average fraction of 80S ribosome occupancy in a window surrounding 3′UTR AUG codons (in all frames) normalized to ORF ribosome density level. **e** Average 40S ribosome occupancy in a window surrounding 3′UTR AUG codons. A peak above background level is evident for the *tma20*∆ and *tma22*∆ strains on AUG codons, consistent with the mechanism of 40S reinitiation. nt nucleotide, rpm reads per million, ORF open reading frame. Source data are provided as a Source Data file. See also Supplementary Fig. 5.

We next investigated the individual contributions of Tma20, Tma22, and Tma64 to recycling by using both 80S and 40S ribosome profiling. Comparison of the average 80S read density around stop codons revealed a queued ribosome peak, the indirect signature of a 40S recycling defect, in data from both the *tma20*∆ and *tma22*∆ strains (Fig. 6a, top). Consistent with this, the average 40S ribosome profiling data revealed a large peak on stop codons in both of these strains (Fig. 6a, bottom). In both cases, the height of the peak was less than that in the respective double knockout strain where *TMA64* was also absent. These data therefore suggest that loss of either Tma20 or Tma22 eliminates most of the 40S recycling function in the cell but that Tma64 may carry out a small fraction of it. This view is consistent with the existence of a small 40S recycling defect in both 40S and 80S data from the *tma64*∆ strain (Fig. 6a, right panels).

We then investigated whether there was gene specificity for any of the Tma proteins. To carry out this analysis, we computed 40S stop codon peak heights for each gene and compared these data for the individual deletion strains against the WT strain (Fig. 6b). As expected, the peaks were highest in the *tma20*∆ and *tma22*∆ strains (Fig. 6b, dots shifted above diagonal). In addition, the peak heights were broadly correlated across knockout strains, supporting the idea that these factors promote 40S recycling on nearly all genes, regardless of the underlying recycling efficiency of a particular stop codon in WT cells. Comparison of 40S stop codon peak heights between *tma20*∆ and *tma64*∆, and *tma22*∆ and *tma64*∆ showed similarly increased peak height for the *tma20*∆ and *tma22*∆ strains (Supplementary Fig. 5a,b), and comparison of peak heights between *tma20*∆ and *tma22*∆ found that the peaks were highly correlated (Supplementary Fig. 5c), again consistent with Tma20/Tma22 being responsible for the majority of 40S recycling activity in the cell. We also examined whether increases in stop codon peaks in the individual deletion strains were differentially modulated by the identity of the penultimate codon. While the codon dependence was very similar for Tma20 vs Tma22 (Supplementary Fig. 5d), the dependence was not well correlated for Tma64 vs Tma20 or Tma22 (Supplementary Fig. 5e). This suggests that the low level of recycling activity exhibited by Tma64 may not be influenced in the same way by the residual tRNA.

Next, we investigated whether there was any specificity to the increased 3′UTR reinitiation phenotype that we had previously shown in the *tma*∆∆ strains^10^. We therefore compared the relative translation levels across small ORFs in 3′UTRs by computing the ratio between 80S ribosome footprint density in 3′UTRs and main ORFs for each of the individual knockout strains (Fig. 6c). In the *tma20*∆ and *tma22*∆ strains, we noted a substantial increase in relative ribosome density in 3′UTRs but only a slight increase in the *tma64*∆ strain. These results are consistent with the effects of 40S recycling failure and reinitiation being linked for these proteins.

We further investigated whether reinitiation in the 3′UTR occurs via an AUG-dependent mechanism for each individual knockout strain. We previously found that much of the reinitiation in the *tma*∆∆ strains required an AUG start codon^10^. We tested this by averaging both 80S and 40S footprints around 3′UTR AUG triplets and normalizing it to the level of translation of the respective main ORF (Fig. 6d and e). In both plots compared to WT cells, we observed an increase in the peak in the *tma20*∆ and *tma22*∆ strains, but not in the *tma64*∆ strain, consistent with the view that their loss accounts for most of the reinitiation in the *tma*∆∆ strains, and further confirming that reinitiation specifically occurs when unrecycled 40S subunits continue into 3′UTR regions and reach AUG codons.

As for the double deletion strains, we were able to detect 40S peaks at 3′UTR stop codons in averaged 40S data for the *tma20*∆ and *tma22*∆ single deletions (Supplementary Fig. 5f). This finding highlights further that Tma20/Tma22 handles most of the recycling burden generally at stop codons.

### Autism mutant impairs 40S ribosome recycling

**Fig. 7:**
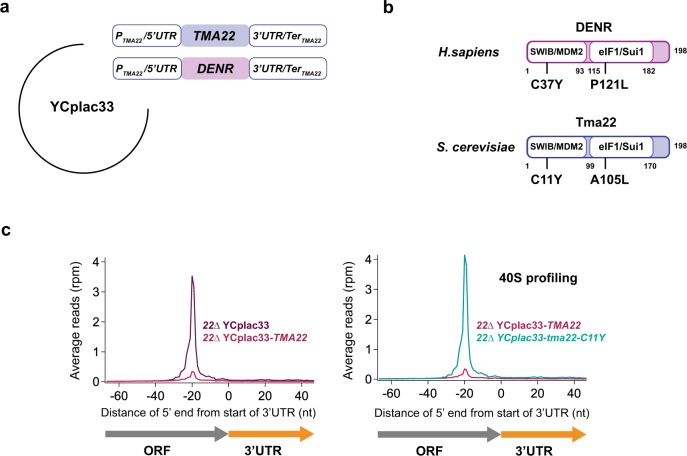
Introduction of an autism-associated mutation into *TMA22* impairs 40S recycling at stop codons. **a** Schematic diagrams of the constructs used to express *S. cerevisiae TMA22* and *H. sapiens DENR* in yeast. The *TMA22* gene along with its natural promoter 5′UTR, 3′UTR, and transcription termination sequences was cloned into the single-copy yeast vector YCplac33. To create a yeast *DENR* expression vector, the coding sequence of the *TMA22* was replaced with *DENR* so that expression would be under the control of the *TMA22* promoter. **b** Schematic representation of the *H. sapiens* DENR and *S. cerevisiae* Tma22 proteins showing homologous domains and the locations of ASD-associated mutations. **c** Average 40S ribosome footprint occupancy from all genes aligned by their stop codons shows complementation of the 40S recycling defect in *tma22*∆ upon expression of WT *TMA22* (left) and loss of recycling activity upon introduction of the C11Y mutation into *TMA22* (right). Footprints plotted by 5′ ends. nt nucleotide, rpm reads per million. Source data are provided as a Source Data file. See also Supplementary Fig. 6.

Finally, we investigated the effect on ribosome recycling of mutations in *DENR* that are associated with autism^22,23^. To test these mutations in yeast, we first developed a single-copy yeast expression plasmid YCplac33^50^ encoding the sequence for the yeast *TMA22* gene or the orthologous human *DENR* gene (Fig. 7a). We then used site-directed mutagenesis to separately introduce the known mutations, C37Y and P121L, into *DENR*. We also introduced the orthologous mutations C11Y and A105L into the *TMA22* expression vector (Fig. 7b). We then performed 40S ribosome profiling on *tma22*∆ strains that had been transformed with these vectors. Compared to an empty vector control, the vector expressing a WT copy of *TMA22* rescued the recycling defect by nearly eliminating the average 40S peak on stop codons (Fig. 7c left). The residual peak is likely due to small changes in expression due to the vector. In contrast, we found that *DENR* could not rescue the phenotype (Supplementary Fig. 6a), despite the protein being detectable by western blot (Supplementary Fig. 6b). This result stands in contrast to previous data showing that MCT-1 could rescue phenotypes associated with loss of *TMA20*^56^ and likely results from lower protein sequence conservation (Supplementary Fig. 6c, 48% identity between Tma20 and MCT-1 vs 34% identity between Tma22 and DENR). Moreover, Tma22 lacks the first 30 amino acids of the SWIB/MDM2 domain in human DENR (Supplementary Fig. 6c).

We then performed ribosome profiling on *tma22*∆ strains containing the vectors where the *TMA22* gene had been mutated. We found that the *tma22*-*A105L* strain did not exhibit a recycling defect, likely due to the lack of conservation around the region of this mutation (Supplementary Fig. 6d). However, the *tma22*-C11Y strain fully recapitulated the recycling defect observed in the *tma22*∆ background (Fig. 7c) and therefore suggests the disease mutation is linked to defective ribosome recycling.

When we expressed the *tma22-C11Y* or *denr-C37Y* mutant, we found protein levels of Tma22 or DENR to be absent or severely reduced, respectively (Supplementary Fig. 6b). Since the C11Y or C37Y mutation is predicted to eliminate a key zinc-mediated interaction between Tma22/Tma20 or DENR/MCT-1, respectfully^24^, it is possible that the absence of this interaction leads to rapid degradation of the Tma22 or DENR protein.

## Discussion

We have utilized a 40S ribosome footprinting approach to directly detect and quantify 40S ribosomal subunits in vivo. Using our method, we found that the majority of 40S footprints in WT cells mapped to start codons, with only a small percentage mapping to stop codons (Fig. 1a), indicating that the kinetics of 60S subunit association during initiation are much slower than 40S subunit dissociation during recycling. As expected, reduction of the pool of 60S subunits by deletion of *RPL11B* resulted in an increase in the relative number of 40S footprints mapping to start codons (Figs. 1a and 2a). Most 40S footprints were similar in length to 80S ribosome footprints with variation in footprint length occurring mainly at the 3′ end, unlike 80S footprints (Fig. 1b and c). Our data indicated, however, that longer footprints were prevalent in 5′UTRs and changes in this distribution in the *rpl11b*∆ strain suggest these correspond to 40S subunits in early phases of initiation before the 40S subunit is competent to join with a 60S subunit (Fig. 1c). In particular, we showed that enhanced peaks on start codons in the *rpl11b*∆ strain allowed us to identify usage of alternative start codons (Fig. 2c and Supplementary Fig. 2). Unlike 80S footprint peaks, 40S peaks offer the distinct advantage of being able to resolve multiple initiation events in noisy environments where multiple ORFs are translated.

We also used the 40S profiling method to directly test the hypothesis that Tma64, Tma20, and Tma22 are 40S ribosome recycling factors^10^. We previously found an increase in 80S ribosome queuing upstream of stop codons in *tma*∆∆ strains but were unable to show what obstacle was blocking their progress. Using 40S profiling, we found an increase in the fraction of 40S footprints mapping to stop codons for both double deletion strains (Fig. 4a) and an increase in the strength of 40S peaks on stop codons (Fig. 5a and b). These findings establish that these Tma proteins recycle 40S subunits at mRNA stop codons in vivo. Without these proteins, the 40S subunits persist on stop codons and eventually exchange the deacylated elongator tRNA in the P-site for ternary complex containing Met-tRNA_i_^Met^. Then, we propose they reinitiate scanning in search of a start codon in 3′UTRs, consistent with previous data showing reinitiation in 3′UTRs^10^ and results here showing increased 40S subunits in 3′UTRs of both double deletion strains (Figs. 4a and 5e-f). Whether the exchange of tRNA is passive or actively catalyzed by a specific protein in these cases remains a question for future investigation. In addition, our observation that 80S ribosomes can be arrested by stalled 40S subunits raises the question of whether these collisions are specifically recognized by the cell, much as collisions between 80S ribosomes are known to be sensed^53^. We previously showed that at least some of the reinitiation in the 3′UTR is AUG-dependent^10^ but were unable to determine if it was due to a reinitiating 40S subunit, 80S ribosome, or both. Here we were able to show 40S ribosome density peaks at 3′UTR AUG codons in both *tma*∆∆ strains (Fig. 5e) and *tma* single deletion strains (Fig. 6e). This observation therefore establishes that an unrecycled 40S subunit can scan into the 3′UTR and reinitiate translation at an AUG codon. While our previous observation that AUG codons are not essential for reinitiation in some cases suggests that 80S reinitiation could also play a role, a 40S scanning process clearly takes place in many cases.

Studies in higher eukaryotes have suggested that orthologs of these Tma proteins could help promote reinitiation downstream of very short uORFs with start codons in good context, particularly those in the ATF4 5′UTR^14–17,29,57^. While our data in yeast suggest these proteins serve as general 40S recycling factors, these alternative roles could occur in specific contexts that allow these proteins to promote recycling of tRNA (and thus allow reloading of Met-tRNA_i_^Met^ needed for reinitiation) without removing the 40S subunit. Whether the Tma proteins promote or prevent reinitiation at a given uORF would therefore hinge on how well the 40S subunit remained associated with the mRNA following loss of the elongator tRNA. In such cases, these factors could be considered tRNA recycling factors, rather than 40S (and tRNA) recycling factors.

We also addressed the question of whether Tma64 (eIF2D) or the heterodimer of Tma20 (MCT-1) and Tma22 (DENR) carry out different roles in the cell by using 40S and 80S ribosome profiling on the individual knockout strains. We found that the Tma20/Tma22 heterodimer is responsible for promoting the majority of 40S recycling and limiting 3′UTR reinitiation at stop codons (Fig. 6). These results suggest that Tma64 plays a minor role or perhaps a specialized role under different conditions. Our data also revealed that the identity of the penultimate codon modulates the dependence of a particular recycling event on the Tma factors and therefore supports the idea that interactions of the residual tRNA within the post-termination 40S subunit may be important. A related study based on ribosome profiling of mammalian cells where DENR was knocked out was recently reported, further demonstrating these factors are important for ribosome recycling and that penultimate codon identity modulates this function^29^.

Last, we explored the connection between ribosome recycling and known DENR mutants that are associated with autism. The introduction of the orthologous autism-associated mutation, C11Y, into *TMA22* (equivalent to the C37Y mutation in *DENR*^22^) resulted in loss of 40S recycling activity (Fig. 7c), likely due to protein destabilization. This finding therefore suggests a linkage between ribosome recycling and ASD. We anticipate that 40S and 80S ribosome profiling of neuronal cell lines where *DENR* is either knocked down or mutated have the potential to provide additional insights into the disease etiology.

## Methods

### Yeast strains

Yeast strains used in this study are listed in Supplementary Table 2. All *Saccharomyces cerevisiae* strains used in this study are derived from the BY4741 background. They were maintained on either YPD plates or SC-Ura plates for transformants.

### Plasmid constructions

Plasmids used in this study are listed in Supplementary Table 3. The primers used for plasmid construction are listed in Supplementary Table 4. Plasmid pDY213 (sc *GRX2*) was constructed by PCR amplifying the *GRX2* gene from yeast genomic DNA using YXp-GRX2f and YXp-GRX2r, digesting with EcoRI-HF and BamHI-HF and inserting the resulting restriction fragment between the EcoRI-HF and BamHI-HF sites of YCplac33. The *GRX2* insert contains 241 nt of sequence upstream of the ORF and 99 nt downstream of the ORF. Plasmid pDY217 (sc *GRX2-FLAG*) was constructed from pDY213 using the Q5 Site-directed mutagenesis kit (NEB; E0554S) and primer pair GRX2_FLAGf / GRX2_FLAGr. Plasmids pDY223 (sc *GRX2-FLAG-M1K*) and pDY225 (sc *GRX2-FLAG-L19A*) were constructed from pDY217 using the Q5 Site-directed mutagenesis kit (NEB; E0554S) and primer pairs GRX2-M1Kf / GRX2-M1Kr and GRX2-L19Af / GRX2-L19Ar respectively.

Plasmid pDY14 (sc *TMA22*) was constructed by PCR amplifying the *TMA22* gene from yeast genomic DNA using TMA22-YXpf and TMA22-YXpr, digesting with EcoRI-HF and BamHI-HF and inserting the resulting restriction fragment between the EcoRI-HF and BamHI-HF sites of YCplac33. The *TMA22* insert contains 235 nt of sequence upstream of the ORF and 295 nt downstream of the ORF. Plasmids pDY135 (sc *tma22-C11Y*) and pDY137 (sc *tma22-A105L*) were constructed from pDY14 using the Q5 Site-directed mutagenesis kit (NEB; E0554S) and primer pairs TMA22-C11Y_SDMf/TMA22-C11Y_SDMr and TMA22-A105L_SDMf/TMA22-A105L_SDMr respectively.

Plasmid pDY153 (sc *DENR*) was constructed using the NEBuilder HiFi DNA Cloning Kit (NEB; E5520S). Briefly, the YCplac33 backbone, along with the upstream 235 nt and downstream 295 nt of *TMA22*, were PCR amplified from pDY14 (sc *TMA22*) using primers YCplac33-Tma22_fwd and YCplac33-Tma22_rev. The *DENR* ORF was PCR amplified from human cDNA using primers DENR_fwd and DENR_rev. The two PCR products were purified and assembled according to the manufacturer’s instructions. Correct integration of the *DENR* ORF into the YCplac33 backbone was confirmed by DNA sequencing. To rule out the chance of errors in the YCplac33 backbone, the *DENR* insert was subcloned from pDY153 (sc *DENR*) by digesting with EcoRI-HF and BamHI-HF and inserting the resulting restriction fragment between the EcoRI-HF and BamHI-HF sites of YCplac33 to produce plasmid pDY155 (sc *DENR* [subcloned]). Plasmids pDY179 (sc *denr-C37Y*) and pDY181 (sc *denr-P121L*) were constructed from pDY155 using the Q5 Site-directed mutagenesis kit (NEB; E0554S) with pDY155, and primer pairs DENR-C37Y_SDMf/DENR-C37Y_SDMr and DENR-P121L_SDMf/DENR-P121L_SDMr, respectively.

Plasmids pDY207 (sc *TMA22-FLAG*), pDY209 (sc *tma22-FLAG-C11Y*) and pDY211 (sc *tma22-FLAG-A105L*) were constructed from plasmids pDY14, pDY135, and pDY137, respectively, using the Q5 Site-directed mutagenesis kit (NEB; E0554S) and primer pair TMA22-FLAGf / TMA22-FLAGr. All plasmids were confirmed by DNA sequencing.

### Ribosome profiling

The ribosome profiling datasets generated for this paper, including number of reads and alignment statistics, are described in Supplementary Table 5. Previously published ribosome profiling datasets used in this paper are listed in Supplementary Table 6.

### 40S ribosome profiling

*Growth of yeast for 40* *S ribosome profiling* — BY4741 (WT), 4715 (*rpl11b*∆), YDY10 (*tma64*∆/*tma20*∆), YDY12 (*tma64*∆/*tma22*∆), 328 (*tma20*∆), 6812 (*tma22*∆), and 4051 (*tma64*∆) were grown in YPD. 6812 (*tma22*∆) transformed with YCplac33 (empty vector), pDY14 (sc *TMA22*), pDY155 (sc *DENR*), pDY135 (sc *tma22-C11Y*), and pDY137 (sc *tma22-A105L*), were grown in SC-Ura. Cells were grown in 1200 mL of media in a 6 L flask to a final OD_600_ of 1.5.

*Formaldehyde cross-linking* — For each culture, 600 mL of cells was poured into two 1 L precooled centrifuge bottles containing 150 g of ice and 16.6 mL of 37% formaldehyde (Sigma; 252549). The centrifuge bottles were mixed by inversion and placed on ice for 1 h, with mixing every 15 min. Cross-linking was stopped by the addition of 30 mL of 2.5 M Glycine (Sigma; G7126). The cells were centrifuged at 3700 × *g* for 20 min at 4 °C, resuspended in 10 mL of water, and combined into one 50 mL conical tube. The cells were centrifuged at 3700 × *g* for 5 min at 4 °C and resuspended in 2 mL of lysis buffer (20 mM Tris pH8, 140 mM KCl, 1.5 mM MgCl_2_, 1% Triton X-100). The resuspended cells were beaded into liquid nitrogen, transferred to a pre-chilled 50 mL conical tube, and stored in a −80 °C freezer.

*Preparation of 40* *S footprint libraries* — Cells were lysed in a Retsch Cryomill (Retsch 20.749.0001). The milled cells were transferred to a 50 mL conical tube, thawed, and spun at 3000 × g for 5 min at 4 °C. The supernatant was transferred to 1.5 mL Eppendorf tubes and spun at full speed for 10 min in a refrigerated benchtop centrifuge at 4 °C. The clarified supernatant was divided into aliquots before being snap frozen in liquid nitrogen and stored at −80 °C.

Lysates were digested with 15 U of RNase I (Ambion; AM2294) per OD for 1 h at room temperature (25 °C), loaded onto 7.5–30% sucrose gradients, and spun at 288,000 x g for 4 h and 45 min at 4 °C in an ultracentrifuge. These gradients offer better separation of the 40S peak than standard 10–50% gradients. The sucrose gradients were fractionated using a Brandel Density Gradient Fractionation System and the isolated 40S peaks were snap frozen in liquid nitrogen and stored at −80 °C.

RNA was purified from the 40S fractions using hot phenol chloroform, with an extended initial incubation of 1 h at 65 °C to reverse the formaldehyde cross-links. 15–80 nt ribosome footprints were size selected (see Supplementary Table 7 for size standards) from a 15% TBE-Urea gel.

Illumina sequencing libraries for a 40S ribosome profiling pilot experiment of BY4741 (WT), 4715 (*rpl11b*∆), YDY10 (*tma64*∆/*tma20*∆), and YDY12 (*tma64*∆/*tma22*∆) were constructed using the method originally described in Ingolia et al., 2009^30^. Sequencing libraries for the replicate libraries of these four strains and all other 40S profiling experiments were performed using a modified protocol based on the updated ribosome profiling method described in McGlincy and Ingolia, 2017^31^.

*Original protocol (based on Ingolia et al., 2012*^58^*)* — The purified RNA fragments were dephosphorylated using PNK (NEB; M0201L) and ligated to universal miRNA cloning linker (NEB; S1315S) using truncated T4 RNA ligase 2 (NEB; M0242L). The ligated footprints were size selected on a 10% TBE-Urea gel, and ribosomal RNA footprints were removed using the Ribo-Zero Gold rRNA Removal Kit Yeast (Illumina; MRZY1306). The ligated footprints were reverse transcribed using the RT primer NI-NI-9 (Supplementary Table 7) and Superscript III (Invitrogen; 18080044). The reverse transcribed footprints were separated from unutilized RT primer on a 10% TBE-Urea gel and circularized using CircLigase ssDNA Ligase (Biosearch Technologies; CL4115K). The circularized libraries were amplified by PCR using Phusion DNA Polymerase (ThermoFisher Scientific; F530L) to add unique 6 nt (“Illumina”) barcodes for index sequencing and common Illumina primer and flow cell binding regions. Library quality and concentration was assessed by BioAnalyzer using the High Sensitivity DNA Kit (Agilent; 5067-4626). Libraries were pooled, and sequencing was performed on an Illumina HiSeq2500 machine at the NIDDK Genomics Core at NIH (Bethesda, MD).

*Modified protocol (based on McGlincy and Ingolia, 2017*^31^*)* — The purified RNA fragments were dephosphorylated using PNK (NEB; M0201L) and ligated to pre-adenylated linkers (Supplementary Table 7) containing a randomized 5 nt Unique Molecular Index (UMI) and a 5 nt sample barcode unique for each sample using truncated T4 RNA ligase 2 (K227Q) (NEB; M0351L). The linkers were pre-adenylated using a 5′ DNA adenylation kit (NEB; E2610L). Unligated linker was removed from the ligation reaction by addition of 5′ deadenylase (NEB; M0331S) and RecJ exonuclease (Biosearch Technologies; RJ411250). Ligated samples were pooled and purified using an oligo clean & concentrator kit (Zymo Research; D4060). Ribosomal RNA footprints were removed from the pooled samples using the Ribo-Zero Gold rRNA Removal Kit Yeast (Illumina; MRZY1306). The pooled samples were reverse transcribed using the RT primer NI-802 (Supplementary Table 7) containing a randomized 2 nt UMI, and Superscript III (Invitrogen; 18080044). The reverse transcribed footprints were separated from unutilized RT primer on a 10% TBE-Urea gel and circularized using CircLigase ssDNA Ligase (Biosearch Technologies; CL4115K). PCR was carried out as described above, adding a 6-nt (Illumina) barcode to be read during the indexing step of the sequencing run. Libraries were assessed and quantified by BioAnalyzer. Sequencing was performed on an Illumina HiSeq3000 machine at the NHLBI DNA Sequencing and Genomics Core at NIH (Bethesda, MD).

### 80S ribosome profiling

*Growth of yeast for 40* *S ribosome profiling* — 4715 (*rpl11b*∆), 328 (*tma20*∆), 6812 (*tma22*∆), and 4051 (*tma64*∆) were grown in YPD. Cells were grown in 750 mL of media in a 2 L flask to a final OD_600_ of 0.6, fast filtered, and frozen in liquid nitrogen.

*Preparation of 80* *S footprint libraries* — Cells were lysed with a Retsch Cryomill (Retsch 20.749.0001) in the presence of frozen lysis buffer (20 mM Tris [pH 8], 140 mM KCl, 1.5 mM MgCl_2_, 1% Triton X-100) containing 0.1 mg/ml cycloheximide (Sigma; C7698). The milled cells were transferred to a 50 mL conical tube, thawed, and spun at 3000 × g for 5 min at 4 °C. The supernatant was transferred to 1.5 mL Eppendorf tubes and spun at full speed for 10 min in a refrigerated benchtop centrifuge at 4 °C. The clarified supernatant was divided into aliquots before being snap frozen in liquid nitrogen and stored at −80 °C.

Lysates were digested with 15 U of RNase I (Ambion; AM2294) per OD for 1 h at room temperature (25 °C), loaded onto 10–50% sucrose gradients, and spun at 274,000 x *g* for 3 h at 4 °C in an ultracentrifuge. The sucrose gradients were fractionated using a Brandel Density Gradient Fractionation System and the isolated 80S peaks were snap frozen in liquid nitrogen and stored at −80 °C. RNA was purified from the 80S fractions using hot phenol chloroform, and 25–34 nt ribosome footprints were size selected (see Supplementary Table 7 for size standards) from a 15% TBE-Urea gel.

Illumina sequencing libraries for 4715 (*rpl11b*∆), 328 (*tma20*∆), 6812 (*tma22*∆), and 4051 (*tma64*∆) were performed using the modified protocol described in McGlincy and Ingolia, 2017^31^ (see above).

### Computational analysis

*Read processing* — Read analysis and sequence alignment were performed as previously described^7,10^ for footprint samples prepared using the protocol based on Ingolia et al., 2012^58^. Briefly, fastq files (debarcoded by the core facility) were trimmed of their linkers using CUTADAPT and footprints measuring 25–34 nt were retained for 80S profiling and 15–80 nt for 40S profiling. Then contaminating tRNA and rRNA were removed with a BOWTIE alignment, allowing two mismatches, to an improved index of noncoding RNAs combining the Saccharomyces genome database RNA gene sequence file (http://downloads.yeastgenome.org/sequence/S288C_reference/rna/archive/rna_coding_R64-1-1_20110203.fasta.gz) and the Genomic tRNA Database (http://gtrnadb2009.ucsc.edu/Sacc_cere/) tRNA sequences file^59^. A CCA was appended to the 3′ end of all tRNAs and a T was appended to the 5′end of all RNAs in the file. The extra 5′ T is commonly observed on reads due to the untemplated activity of reverse transcriptase during library creation. The index further included a fragment of the Aspartate tRNA, tD(GUC)K, sequence with two T residues appended to the 5′ end (TTTCCGTGATAGTTTAATGGTCAGAATGGGC) that was found to be heavily overrepresented in our datasets. While this sequence was the biggest contributor, we noted small contributions from other tRNA fragments with untemplated bases added to the 5′ ends, particularly at very short (<20 nt) length scales.

Following removal of these ncRNA sequence, the resulting fastq files were aligned to the genome and then splice junctions using BOWTIE, allowing one mismatch but no multimapping reads. For 80S ribosome profiling libraries only, reads that failed to align were trimmed of poly(A) on their 3′ ends and remapped to ensure that ribosomes that had partially read into poly(A) tails were not eliminated. This step prevents loss of footprints derived from ribosomes that reinitiate in 3′UTRs and partially protect the poly(A) tail. We used BOWTIE version 1.1.2 or 1.01^60^ and included the parameter -y for all runs. We used the parameters -a -m 1 --best --strata for alignments to coding regions only.

A slightly modified protocol was used for libraries generated using the method of McGlincy and Ingolia, 2017^31^. Fastq files were trimmed of their linkers and separated according to their 5-nt internal sample barcode by using CUTADAPT. Contaminating tRNA and rRNA were removed with a BOWTIE alignment to the previously described index of noncoding RNAs. Then, all PCR duplicates were removed using a simple python script to compare the 7-nt UMIs. Following removal of the UMIs with CUTADAPT, the resulting fastq files were aligned to the genome and splice junctions exactly as described above for the original protocol.

Other analysis software used Biopython 1.58 or 1.63. In general, ORFs marked dubious or those that overlapped with other transcripts were ignored in the analysis. Annotations for 3′ UTRs^61^ that used coordinates from the R64-1-1 genome assembly were downloaded from Saccharomyces Genome Database Project. Python code for the three basic analyses (Gene average, gene quantitation, and position-average plots) has been published previously^10^ and is on Github: https://github.com/guydoshlab.

The proportion of 40S footprints in different mRNA regions (Figs. 1 and 4) was determined by first aligning reads that had been first subtracted for rRNA and tRNA and deduplicated to a BOWTIE library of transcripts with spliced coding sequences. Only one mismatch was allowed. Reads were then assigned as mapping to 5′ UTR, start codon, ORF, stop codon, or 3′ UTR. A read was defined to have mapped to a start codon if any portion of it overlapped with a 9 nt region centered around the AUG. A read was defined to have mapped to a stop codon if any portion of it overlapped with a 9 nt region ending with the stop codon at the 3′ end.

For all 40S analysis, except Figs. 1, 4, and S3a (top), and S3b, footprints of 26–42 nt in length were used to enhance precision. When available, replicate datasets were combined to enhance sequencing depth. However, 2-D metagene plots (Figs. 1c and 4c), some *GCN4* analysis (Fig. S3a top and S3b), and examples of N-terminal extension (Fig. 2c and S2) relied on the rep_2 datasets only since individual peaks were sharper and aided analysis.

*Analysis of alignments* — All average analysis of footprint data around annotated main start and stop codons (creating a metagene) was performed by computing the average number of reads (in rpm) at each nucleotide position. This analysis was used for Figs. 1c, 2a, 4c, 5a, 6a, 7c, S1b, and S6a, d. For 2-D metagene plots (Figs. 1c and 4c), this analysis was performed by first separating the mapped footprint data by length and computing the average for each footprint length separately (15–80 nt).We excluded genes with features that were smaller than a window size of ±100 nt of the feature of interest for 2-D metagenes or 100 nt of UTR and 300 nt of ORF for 1-D metagenes).

Quantitation of gene-level footprint occupancy (i.e., counting reads mapping to genes for Figs. 2b, 5b, 6b-c, S4a–c and S5a–c) was performed by creating footprint density in units of reads per kilobase per million mapped reads (rpkm) by taking the reads mapping to an annotated sequence (in rpm units) and dividing by the gene length in kilobases. For footprint gene quantitation analyses of 3′UTR regions, 3′UTRs were extended 25 nt downstream from their annotated endpoints in order to ensure all ribosomes that partially protected poly(A) sequences were accounted for. Reads that mapped in the first or last 15 nt of ORFs were left out to eliminate artefacts associated with initiating and terminating ribosomes. To be included in the analysis, a threshold of 5 rpkm was required of ORF reads and 0.5 rpkm for 3′UTR reads, unless noted otherwise. 80S footprint reads were shifted by 13 nt and 40S footprint reads were shifted by 14 nt to correspond to the P site. mRNA-Seq reads were unshifted. Correlation analysis of 3′UTR:ORF ratios (Fig. 6c) was done with a required threshold of 5 rpkm in ORFs and 0.5 rpkm in 3′ UTRs. Quantitation of peak reads around start or stop codons was performed by summing reads within a 5 nt region around the peak. Pearson *R*^2^ was computed on log-transformed data.

Position-average (metacodon) plots (Figs. 3b, 5e-f, 6d-e, S4e, and S5f) were similarly created by averaging together reads in a window around every occurrence of a particular motif in a UTR region (such as start or start codon). Genes with no reads in the UTR region were excluded from these analyses. For the analysis in Figs. 6d and S4e, reads were normalized to the total reads present in the ORF and only genes exceeding a threshold of 5 rpkm were used. Genes with 5′ UTR introns were removed from the analysis for position-average plots and quantitation to avoid artifacts.

Analysis of penultimate codon effects (Figs. 5c-d, S4d, S5d-e) on 40S recycling was performed by computing the average 40S stop codon peak ratios between mutant and WT strains for genes with common penultimate codons (Supplementary Data 1). Penultimate codons found at least *N* = 10 times in the transcriptome were used for downstream analysis. To determine the likelihood that the average ratio for a given codon was significantly different from the average for the entire transcriptome, we used bootstrap analysis. From the total distribution of ratios, we selected *N* ratios (with replacement) 20,000 times and computed the confidence interval (percentile range) at which the mean ratio for each penultimate codon occurred.

### Purification of yeast mitochondria

Yeast mitochondria were purified according to the protocol described in Meisinger et al. (2006)^62^ and Gregg et al. (2009)^63^.

*Growth of yeast for purification of yeast mitochondria* — BY4741 (WT) transformed with YCplac33 (empty vector), pDY217 (sc *GRX2-FLAG*), pDY223 (sc *GRX2-FLAG-M1K*), and pDY225 (sc *GRX2-FLAG-L19A*), were grown in 1000 mL of SC- U media in a 6 L flask to a final OD_600_ of 1.5–2.0.

*Isolation of crude mitochondrial fraction* — Cultures were poured into two 500 mL centrifuge tubes and spun at 3000 × g for 5 min at RT. The cells were washed twice with 250 mL of dH_2_O before being resuspended in DTT buffer (100 mM Tris/H_2_SO_4_ pH 9.4, 10 mM dithiothreitol; 2 mL of buffer/g [wet weight] cells). Cells were rotated in a 50 mL centrifuge tube in a shaker at 30 °C for 20 min at 70 rpm. Cells were harvested by centrifugation at 3000 × *g* for 5 min at RT. The cells were washed once with Zymolyase buffer (20 mM potassium phosphate pH 7.4, 1.2 M sorbitol), resuspended in Zymolyase buffer (7 mL of buffer/g [wet weight] cells) and transferred to a 125 mL glass flask. Zymolyase-20T (5 mg of Zymolyase-20T/g [wet weight] cells) was added to the cell suspension and rotated in a shaker at 30 °C for 30 min at 70 rpm. Spheroblasts were pelleted by centrifugation at 2200 × g for 8 min at 4 °C, washed once with ice-cold homogenization buffer (10 mM Tris/HCl pH 7.4, 0.6 M sorbitol, 1 mM EDTA, 0.2% [w/v] BSA), and resuspended in ice-cold homogenization buffer (6.5 mL of buffer/g [wet weight] cells) and transferred to a pre-chilled glass homogenizer. Cells were homogenized with 15 strokes of the pestle, before addition of one volume of ice-cold homogenization buffer, and transferred to a 50 mL centrifuge tube. Unbroken cells, nuclei, and large debris were pelleted by centrifugation at 1500 × *g* for 5 min at 4 °C followed by centrifugation at 3000 × g for 5 min at 4 °C. The supernatant was centrifuged at 12000 × g for 15 min at 4 °C to pellet the crude mitochondria and resuspended in 3 mL of ice-cold SEM buffer (10 mM MOPS/KOH pH 7.2, 250 mM sucrose, 1 mM EDTA).

*Purification of mitochondria from other cellular organelles* — The crude mitochondrial fraction was loaded onto a sucrose gradient composed of 1.5 ml of 60% (w/v) sucrose in EM buffer (10 mM MOPS/KOH pH 7.2, 1 mM EDTA), 4 mL of 32% (w/v) sucrose, 1.5 mL 23% (w/v) sucrose, and 15% (w/v) sucrose (all in EM buffer). The sucrose gradients were centrifuged in a Beckman SW41 Ti swinging-bucket rotor at 134,000 × g for 1 h at 4 °C. The mitochondrial band was recovered from the 60% sucrose/32% sucrose interface and centrifuged in a Beckman benchtop ultracentrifuge at 10,000 × *g* for 30 min at 4 °C. The pelleted purified mitochondria were resuspended in 20% TCA transferred to an Eppendorf tube and stored at −80 °C.

### Western blot analysis

Yeast strains were grown to log-phase before preparation of whole cell extracts (WCEs) from 5 ODs of cells. Protein was prepared from purified mitochondria and WCEs by TCA extraction. Western analysis was conducted using mouse monoclonal antibodies against FLAG (Sigma; F1804), VDAC1/Porin (Abcam; ab110326), DENR (Abnova; H00008562-M01), and beta-actin (Abcam; ab8224). A 1/1000 dilution was used for all primary antibodies. All westerns were repeated at least twice on two independently grown cultures for each condition.

### Quantification and statistical analysis

For western blots, at least two individual replicates were performed to verify the result was reproducible. Graphs were made with Igor Pro 8 (Wavemetrics). Ribosome footprint data were processed with custom Python 2.7 scripts. For most analysis, replicate ribosome profiling runs were generally pooled. However, replicates are displayed separately to support key conclusions.

### Reporting summary

Further information on research design is available in the Nature Research Reporting Summary linked to this article.

## Supplementary information

Supplementary Information

Description of Additional Supplementary Files

Supplementary Data 1

Reporting Summary

## Data Availability

Raw and analyzed data have been deposited in the NCBI GEO database under the accession number GSE145904. Raw and analyzed data for ribosome profiling datasets from previous papers (see Supplementary Table 6) are available from the NCBI GEO database under the accession number GSE108942. The Saccharomyces genome database RNA gene sequence file can be downloaded from the Saccharomyces Genome Database [http://downloads.yeastgenome.org/sequence/S288C_reference/rna/archive/rna_coding_R64-1-1_20110203.fasta.gz] and the Genomic tRNA Database tRNA sequences file is available from the Genomic tRNA database (http://gtrnadb2009.ucsc.edu/Sacc_cere/). Annotations for 3′ UTRs from the R64-1-1 genome assembly were downloaded from the Saccharomyces Genome Database Project [https://sgd-prod-upload.s3.amazonaws.com/S000204042/Nagalakshmi_2008_PMID_18451266_V64_track_files.zip]. Source data are provided with this paper.

## Source data

Source Data

## Code availabilty

Custom code used in this study is available on Github [https://github.com/guydoshlab/Yeastcode1].
