# Supplementary material for: 40S ribosome profiling reveals distinct roles for Tma20/Tma22 (MCT-1/DENR) and Tma64 (eIF2D) in 40S subunit recycling
Source: Nat Commun. 2021 May 20;12:2976. doi: 10.1038/s41467-021-23223-8 (PMC8137927; doi:10.1038/s41467-021-23223-8)
Supplement: Supplementary file 1 — Supplementary Information [file 41467_2021_23223_MOESM1_ESM.pdf]

Supplementary Information for

40S ribosome profiling reveals distinct roles for Tma20/Tma22 (MCT-1/DENR) and Tma64 (eIF2D) in 40S subunit recycling

David J. Young[1], Sezen Meydan[1, 2], and Nicholas R. Guydosh[1*]

[1] Laboratory of Biochemistry and Genetics, National Institute of Diabetes and Digestive and Kidney Diseases, National Institutes of Health, Bethesda, MD 20892

[2] Postdoctoral Research Associate Training Program, National Institute of General Medical Sciences, National Institutes of Health, Bethesda, MD 20892

[*]Corresponding author: nicholas.guydosh@nih.gov (lead contact)

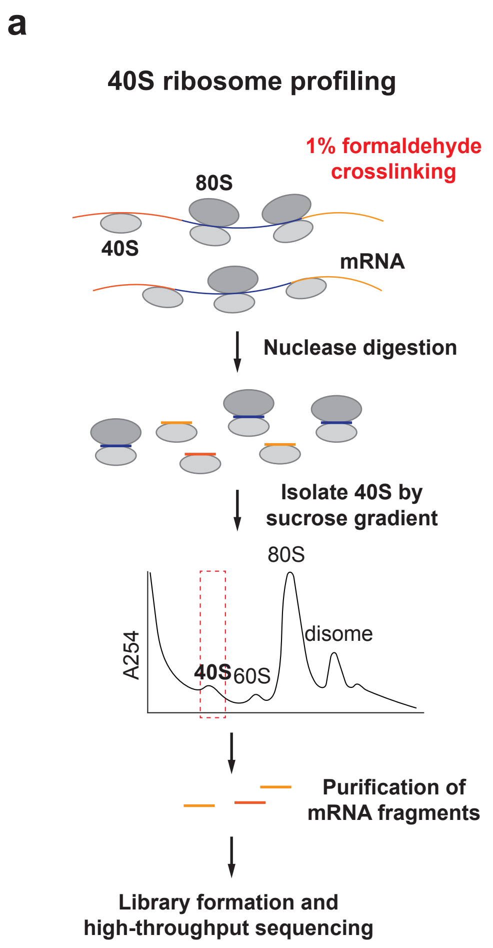

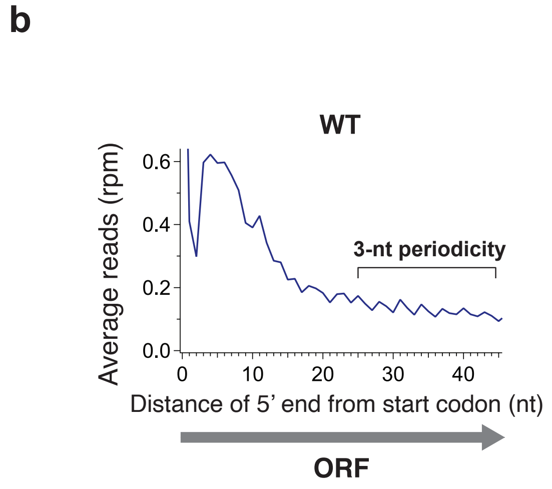

Supplementary Figure 1

Supplementary Figure 1. The 40S ribosome profiling strategy and supplemental assay data. Related to Figure 1.

(a) Cells were cross-linked with 1% formaldehyde to stabilize ribosomal complexes. Cell lysates were digested with RNase I and separated by sucrose gradient fractionation. Footprints were extracted from 40S fractions by acidic phenol/chloroform, size selected, and used in the construction of Illumina sequencing libraries. **(b)** One-dimensional metagene for 40S WT data from all genes aligned at their start codon. Data plotted by 5' footprint ends. A clear signature of 3-nt periodicity can be seen in reads mapping to the ORF. This plot shows a zoomed view of Figure 1c, upper right panel. Abbreviations: nt, nucleotide; rpm, reads per million. Source data are provided as a Source Data file.

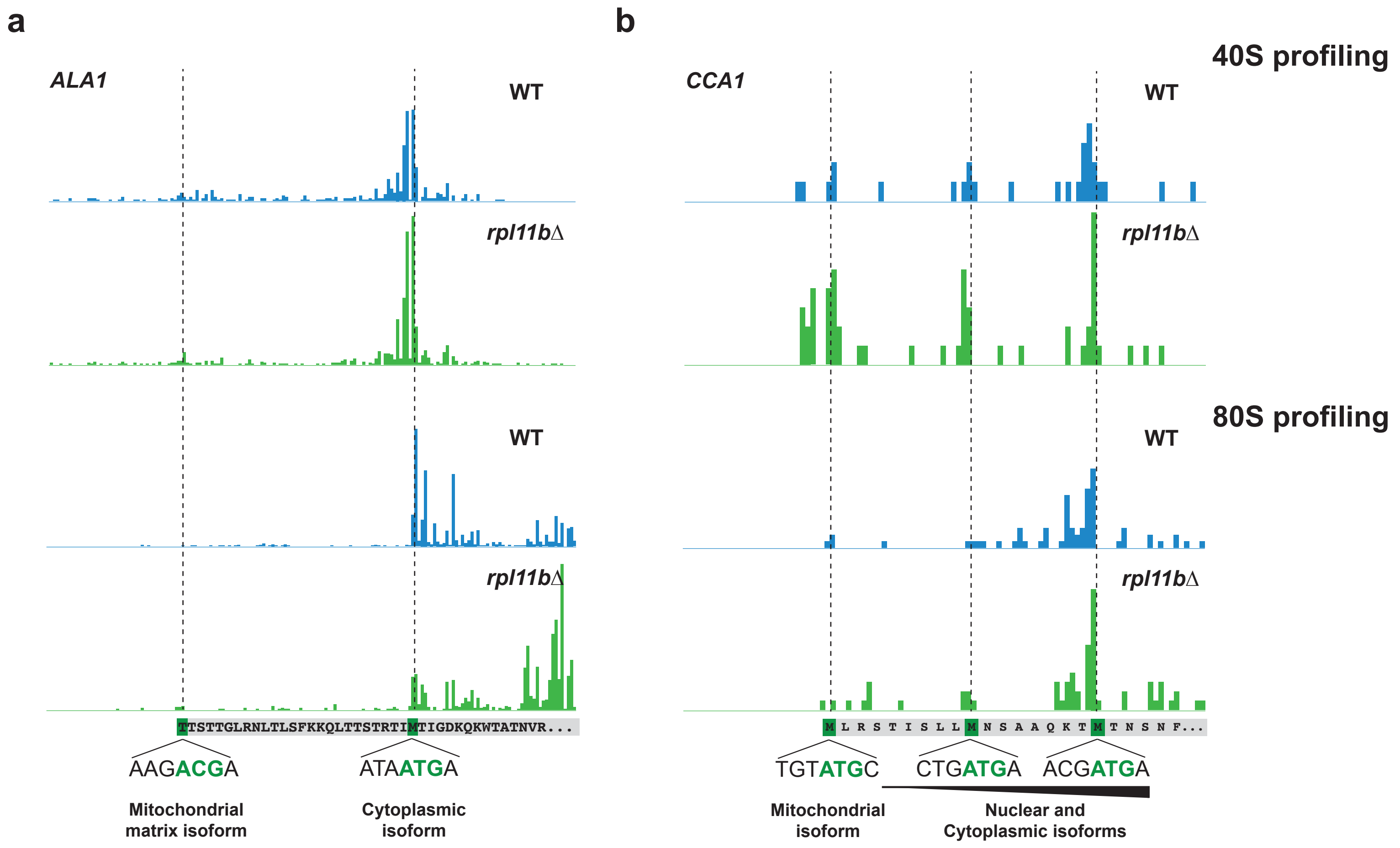

Supplementary Figure 2

Supplementary Figure 2. Multiple 40S occupancy peaks at the 5' end of genes identifies N-terminally extended protein isoforms. Related to Figure 2.

Ribosome footprint profiles of the 5' ends from 40S and 80S ribosome profiling data corresponding to the *ALA1* and *CCA1* genes. The 40S footprint occupancy peaks at cognate AUG and near-cognate ACG codons reveal N-terminal extensions that are also apparent in the 80S data. Gene annotations are drawn to correspond to data for ribosome P sites. Dotted lines correspond to the first position of the P site. DNA sequences (and amino acids encoded by cognate tRNAs) for start codon contexts are shown in the schematic. Source data are provided as a Source Data file.

a

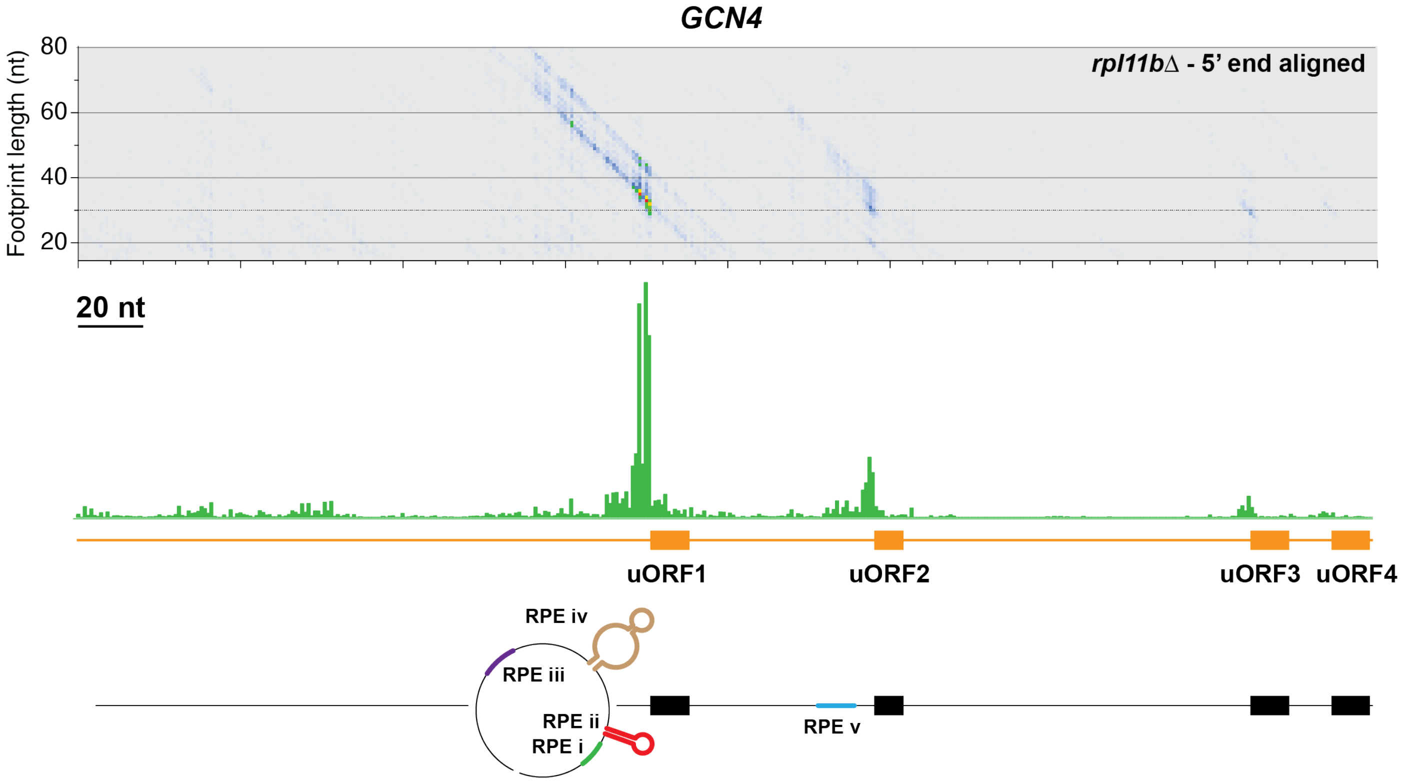

b

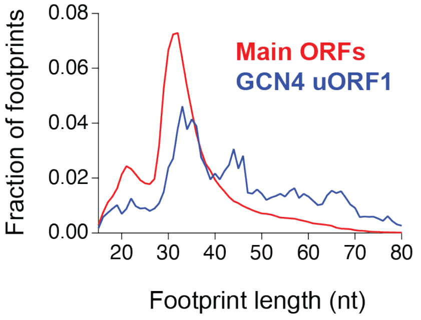

Supplementary Figure 3

Supplementary Figure 3. Analysis of 40S ribosome occupancy at *GCN4* uORF start codons. Related to Figure 3.

(a) (*Top*) 5' end aligned 40S footprints for the 5'UTR of the *GCN4* gene showing increased numbers of longer 40S footprints (>45 nt) at uORF1 and uORF2; the color scale represents the read count. Data plotted as 5' ends for *rpl11b*Δ. (*Middle*) 40S ribosome footprint profile of the 5'UTR of the *GCN4* gene for *rpl11b*Δ showing 40S footprint occupancy peaks at 5'UTR uORF AUG codons. Reads upstream of uORF 1 likely belong to the "nAuORFs." Gene annotations are drawn to correspond to data for ribosome P sites (AUG codons). (*Bottom*) Schematic of reinitiation promoting elements (RPEs) and structures that have been shown to be important for reinitiation at *GCN4* uORF1 and uORF2 (model for RPEs is based on Gunisova et al., 2018 [52]). This shows in a general sense how binding by RPEs from neighboring regions could help extend the 5' end of the protected 40S footprint. **(b)** Footprint sizes for 5' ends mapping between 75 nt upstream to 15 nt downstream of the start codon for *GCN4* uORF1 compared to the same analysis for all main ORF start codons. Data show how footprints at uORF1 are longer than is typical at main ORF start codons. Data shown for *rpl11b*Δ cells. Abbreviations: nt, nucleotide. Source data are provided as a Source Data file.

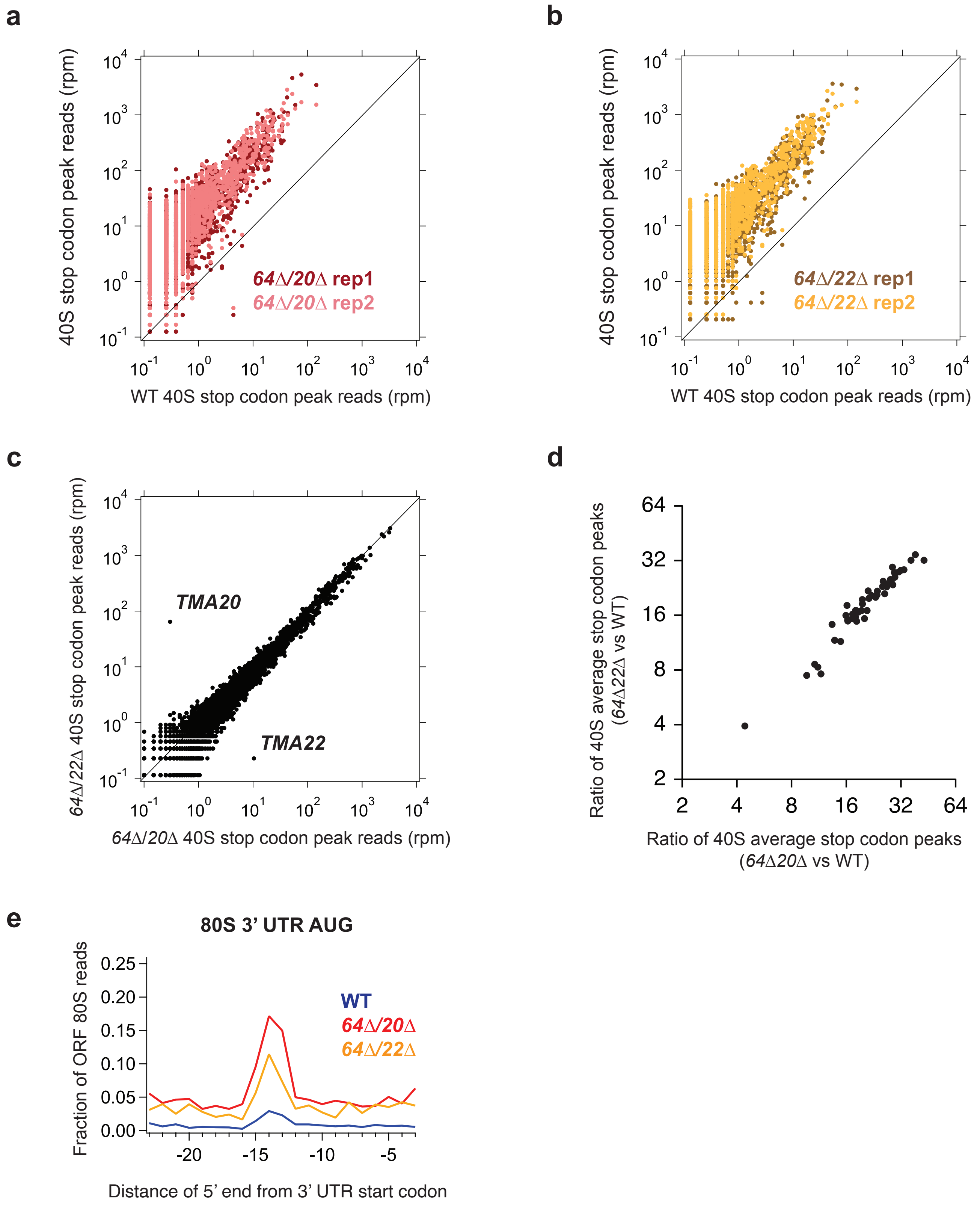

Supplementary Figure 4

Supplementary Figure 4. Replicate analysis of 40S stop codon peak height in the *tma*ΔΔ strains. Related to Figure 5.

(a + b) Correlation analysis of 40S stop codon peak heights (total count of footprints at the stop codon) between the WT strain and replicate datasets for the *tma64*Δ/*tma20*Δ (a) and *tma64*Δ/*tma22*Δ (b) strains. The plots reveal a strong correlation between the replicates of the *tma*ΔΔ strains. **(c)** Correlation analysis of 40S stop codon peak heights between the *tma64*Δ/*tma20*Δ and *tma64*Δ/*tma22*Δ strains. There is a strong correlation between the two *tma*ΔΔ strains, showing each mutant has similar effects on recycling. Each point represents the data for one gene. Footprint reads were quantitated by shifting them by 14 nt and then counting reads around the stop codon. **(d)** Comparison of the average ratio between mutant vs WT 40S stop codon peaks across subsets of penultimate codons for the *tma64*Δ/*tma20*Δ and *tma64*Δ/*tma22*Δ strains. Each dot represents one penultimate codon. Strong correlation implies both strains share similar dependence on penultimate codon identity. **(e)** Average fraction of 80S ribosome occupancy in a window surrounding 3'UTR AUG codons normalized to ORF ribosome occupancy level (all frames included) for WT and *tma*ΔΔ strains. 80S data have been previously published [10]. Abbreviations: rpm, reads per million. Source data are provided as a Source Data file.

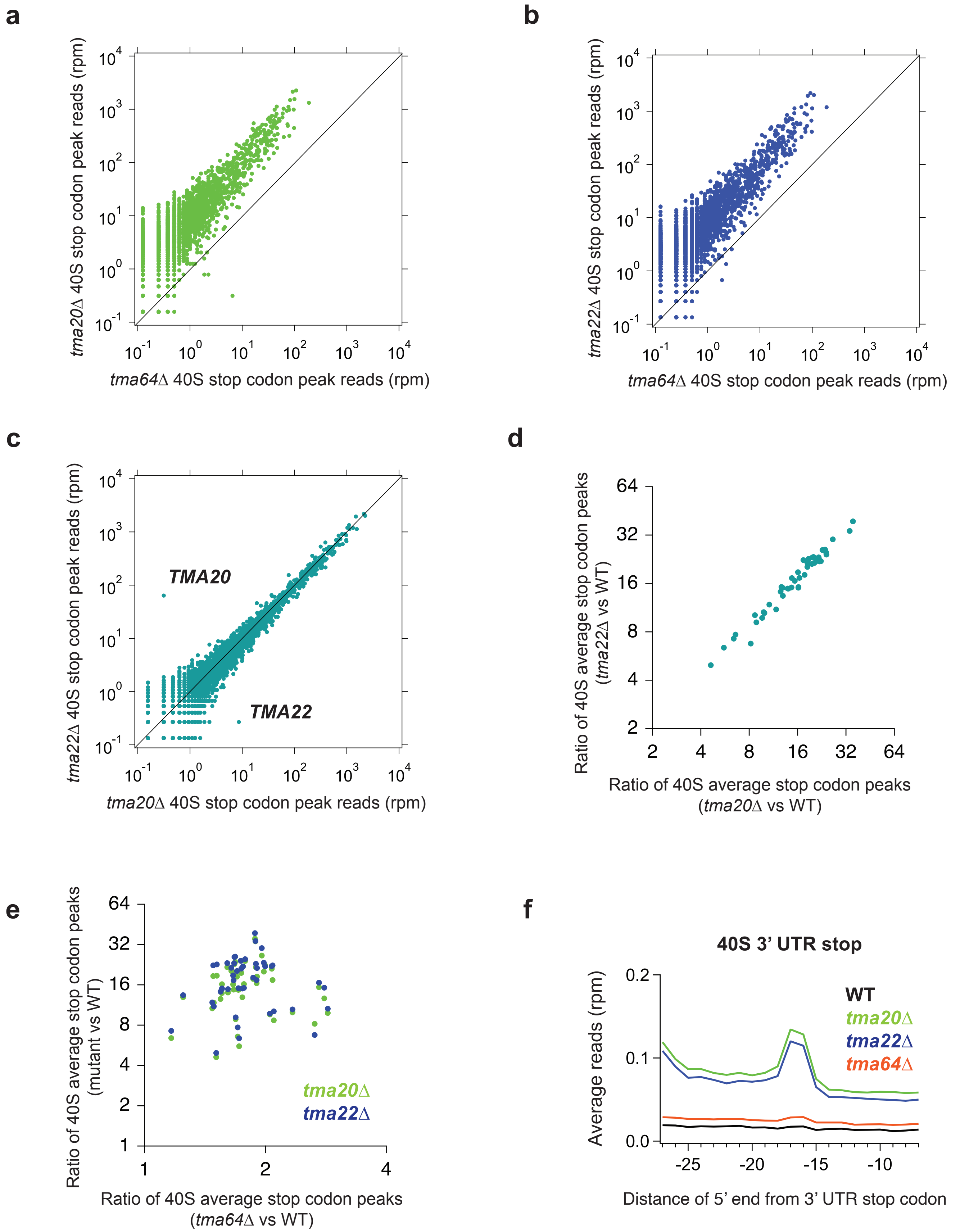

Supplementary Figure 5

Supplementary Figure 5. Correlation analysis of 40S stop codon peak height for *tma20*Δ, *tma22*Δ, and *tma64*Δ. Related to Figure 6.

(a + b) Analysis of 40S stop codon peak heights (total count of footprints at the stop codon) for *tma20*Δ (a) and *tma22*Δ (b) strains, compared to *tma64*Δ. The strong increase in stop codon peak height in the *tma20*Δ and *tma22*Δ strains (dots above the diagonal) indicates that *tma20*Δ and *tma22*Δ are responsible for most of the 40S recycling activity in the cell for nearly all genes. **(c)** Correlation analysis of 40S stop codon peak heights between the *tma20*Δ and *tma22*Δ strains. There is a strong correlation between the two *tma*Δ strains. Each point represents the data for one gene. Footprint reads were quantitated by shifting them by 14 nt and then counting reads around the stop codon. Overall, these data establish that the Tma factors have similar effects on 40S recycling across the transcriptome, though to a lesser extent for Tma64. **(d)** Comparison of the average ratio between mutant vs WT 40S stop codon peaks across subsets of penultimate codons for the *tma20*Δ and *tma22*Δ strains. Each dot represents one penultimate codon. Strong correlation implies both strains share similar dependence on penultimate codon identity. **(e)** Comparison of the average ratio between mutant vs WT 40S stop codon peaks across subsets of penultimate codons for the *tma64*Δ and *tma20*Δ or *tma22*Δ strains. Each dot represents one penultimate codon. Poor correlation implies that Tma64 does not share the same dependence on penultima codon identity as Tma20/22. **(f)** Average 40S ribosome occupancy in a window surrounding 3'UTR stop codons for the *tma* single deletion strains. The peaks in the *tma20*Δ and *tma22*Δ data are consistent with Tma20 and Tma22 carrying out the majority of 40S recycling activity in the cell. Abbreviations: rpm, reads per million. Source data are provided as a Source Data file.

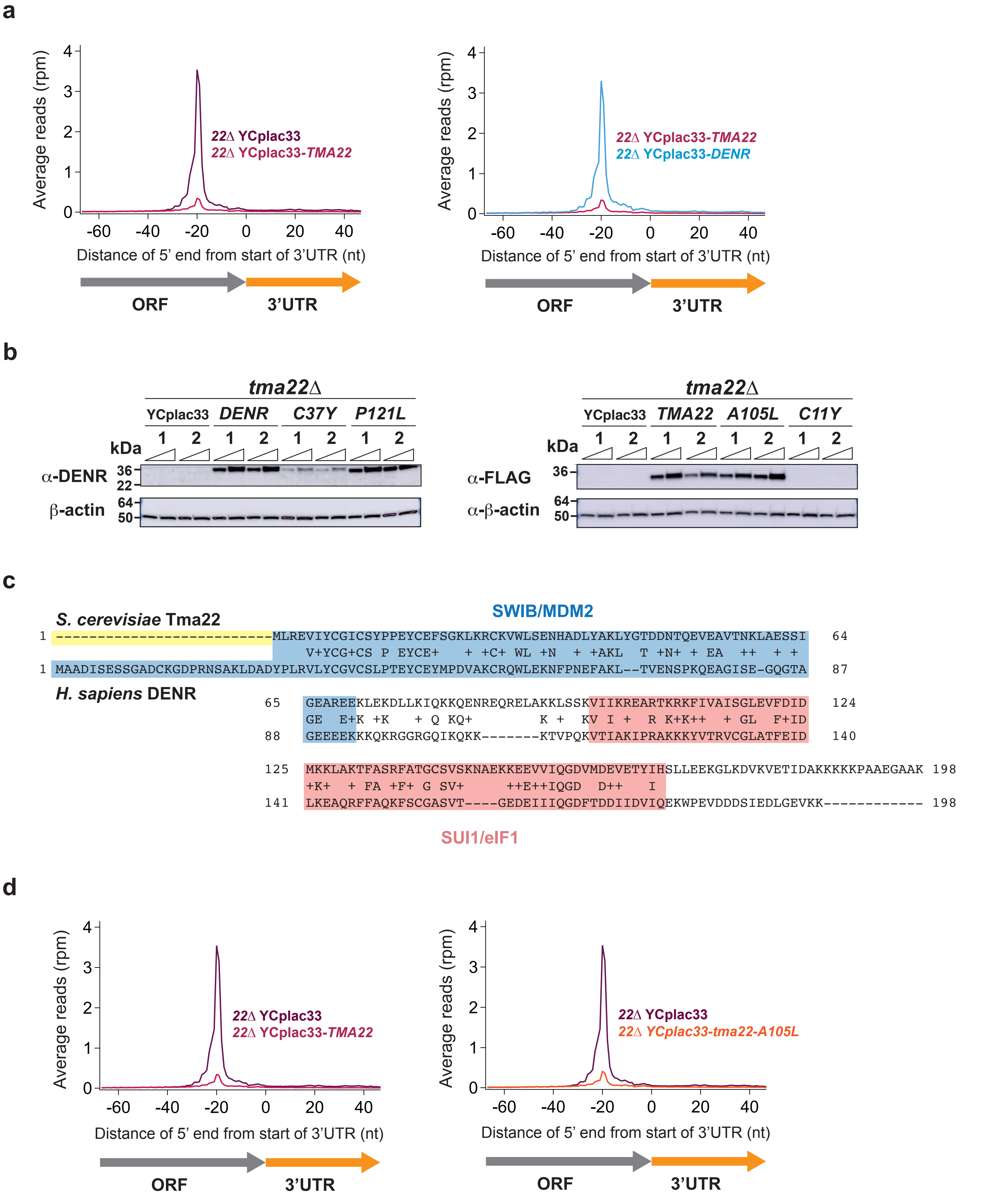

Supplementary Figure 6

Supplementary Figure 6. Autism-associated mutations in TMA22 and DENR. Related to Figure 7.

(a) Average 40S ribosome footprint occupancy for all genes aligned by their stop codons for *tma22*Δ YCplac33, *tma22*Δ YCplac33-*TMA22*, and *tma22*Δ YCplac33-*DENR*, showing that human DENR is unable to complement the 40S recycling defect in *tma22*Δ. Footprints plotted by 5' ends. **(b)** (*left*) Whole cell extracts from *tma22*Δ cells expressing human DENR and human DENR carrying either the C37Y or P121L autism-associated mutations were subjected to western analysis using mouse monoclonal antibodies against DENR (top blot) or β-actin (control, bottom blot). (*right*) Whole cell extracts from *tma22*Δ cells expressing FLAG-tagged Tma22 and FLAG-tagged Tma22 carrying either the C11Y or A105L mutations (equivalent to C37Y and P121L mutations, respectively) were subjected to western analysis using mouse monoclonal antibodies against FLAG (top blot) or β-actin (control, bottom blot). Data are shown for two independent transformants for each plasmid. Two amounts of extracts (1x and 2x) were loaded in each lane pair. Expression of DENR-C37Y and Tma22-C11Y are substantially decreased relative to expression of WT DENR or Tma22 and DENR-P121L or Tma22-A105L, respectively. **(c)** Alignment of the protein sequences of *S. cerevisiae* Tma22 and *H. sapiens* DENR. The conserved SWIB/MDM2 and SUI1/eIF1 domains are shown in blue and apricot (respectively). The N-terminal truncation of the SWIB/MDM2 domain in *S. cerevisiae* Tma22 is highlighted in yellow. **(d)** Average 40S ribosome footprint occupancy for all genes aligned by their stop codons for *tma22*Δ YCplac33, *tma22*Δ YCplac33-*TMA22*, and *tma22*Δ YCplac33-*tma22-A105L*, showing that the A105L mutation does not affect the 40S recycling activity of Tma22. Abbreviations: nt, nucleotide; rpm, reads per million; kDa, kilodalton. Source data are provided as a Source Data file.

Supplementary Table 1: The level of rRNA contamination in 40S profiling libraries.

Sample name	Description	Reads aligned to genome/splice jcns	# of reads processed*	% of reads not aligned to ncRNA
DY134Ffx	WT_1	4,558,820	76,534,682	6.0
DY135Ffx	*rpl11b*Δ_1	4,726,962	59,797,074	7.9
DY136Ffx	*tma64*Δ/*tma20*Δ_1	7,992,061	74,676,100	10.7
DY137Ffx	*tma64*Δ/*tma22*Δ_1	4,839,435	85,808,190	5.6
DY182Ffx	WT_2	5,437,081	51,703,250	10.5
DY183Ffx	*rpl11b*Δ_2	5,555,478	55,413,139	10.0
DY184Ffx	*tma64*Δ/*tma20*Δ_2	11,967,233	82,777,298	14.5
DY185Ffx	*tma64*Δ/*tma22*Δ_2	3,965,553	43,024,579	9.2

* Note: # of reads processed tally for rep_2 samples excludes PCR duplicates

Supplementary Table 2: Yeast Strains used in this study, related to Methods.

Yeast Name	Genotype	Source
BY4741	*MATa his3Δ1 leu2Δ0 met15Δ0 ura3Δ0*	Horizon Discovery
4715	*MATa his3Δ1 leu2Δ0 met15Δ0 ura3Δ0 rpl11bΔ::KanMX4*	Horizon Discovery
YDY10	*MATa his3Δ1 leu2Δ0 met15Δ0 ura3Δ0 tma64Δ::HygMX4 tma20Δ::KanMX4*	Young et al. 2018
YDY12	*MATa his3Δ1 leu2Δ0 met15Δ0 ura3Δ0 tma64Δ::HygMX4 tma22Δ::KanMX4*	Young et al. 2018
328	*MATa his3Δ1 leu2Δ0 met15Δ0 ura3Δ0 tma20Δ::KanMX4*	Horizon Discovery
6812	*MATa his3Δ1 leu2Δ0 met15Δ0 ura3Δ0 tma22Δ::KanMX4*	Horizon Discovery
4051	*MATa his3Δ1 leu2Δ0 met15Δ0 ura3Δ0 tma64Δ::KanMX4*	Horizon Discovery

Supplementary Table 3: Plasmids used in this study, related to Methods.

Plasmid	Description	Source
YCplac33	sc[1] *URA3*	Gietz and Sugino 1988
pDY213	sc *URA3 GRX2* in YCplac33	This study
pDY217	sc *URA3 GRX2-FLAG* in YCplac33	This study
pDY223	sc *URA3 GRX2-FLAG-M1K* in YCplac33	This study
pDY225	sc *URA3 GRX2-FLAG-L19A* in YCplac33	This study
pDY14	sc *URA3 TMA22* in YCplac33	This study
pDY135	sc *URA3 tma22-C11Y* in YCplac33	This study
pDY137	sc *URA3 tma22-A105L* in YCplac33	This study
pDY153	sc *URA3 DENR* in YCplac33	This study
pDY155	sc *URA3 DENR* in YCplac33 (subcloned)	This study
pDY179	sc *URA3 denr-C37Y* in YCplac33	This study
pDY181	sc *URA3 denr-P121L* in YCplac33	This study
pDY207	sc *URA3 TMA22-FLAG* in YCplac33	This study
pDY209	sc *URA3 tma22-FLAG-C11Y* in YCplac33	This study
pDY211	sc *URA3 tma22-FLAG-A105L* in YCplac33	This study

[1]sc, single-copy

Supplementary Table 4: Oligonucleotides used in this study, related to Methods.

Primer No.	Primer Name	Primer Sequence 5' - 3'
**Primers used for plasmid construction**		
DYP1006	YXp-GRX2f	TTTCAGGAATTCCCTTTCCTGTTTCTTCCTCAAGC
DYP1008	YXp-GRX2r	TTTCAGGGATCCATCTAGCTAGAAGTTTTGTAGG
DYP1009	GRX2_FLAGf	gatgacgacaagTAGTTTAAATTACGCTAATATCC
DYP1010	GRX2_FLAGr	gtctttgtagtcTTGAAATACCGGCTTCAATATTTC
DYP1013	GRX2-M1Kf	TATTGTTTCAaaGAGACCAATTTTTCCTTC
DYP1014	GRX2-M1Kr	TATAACAGTAGTTGGAGTG
DYP1023	GRX2-L19Af	CATTATCACGgctTTTGCCACAAGAATTATTG
DYP1024	GRX2-L19Ar	ATAATAACAATTAAATTCGAGTCG
DYP29	TMA22-YXpf	TTTCAGGAATTCCTGACGTGAACTTGCACCATG
DYP30	TMA22-YXpr	TTTCAGGGATCCAACTATACTTACTTACGGCTGG
DYP920	TMA22-C11Y_SDMf	TGTGGAATTTaCTCCTACCCAC
DYP921	TMA22-C11Y_SDMr	ATAGATGACTTCTCTTAACATTTTATG
DYP922	TMA22-A105L_SDMf	TAAAAGAGAAttgAGGACCAAGAGAAAGTTTATC
DYP923	TMA22-A105L_SDMr	ATGATCACTTTCGATGAC
DYP978	YCplac33-Tma22_fwd	GCAATCCATAAATATGTAATAGC
DYP979	YCplac33-Tma22_rev	TTTATGCAATATGCTTTTCTTTAG
DYP980	DENR_fwd	AGAAAAGCATATTGCATAAAATGGCTGCTGACATTTCTG
DYP981	DENR_rev	ATTACATATTTATGGATTGCTCACTTCTTTACTTCTCCAAG
DYP982	DENR-C37Y_SDMf	TGTGGAGTCTaTTCATTACCAAC
DYP983	DENR-C37Y_SDMr	ATAAAGGACTCGAAGTGG
DYP984	DENR-P121L_SDMf	GCCAAAATTCtCAGAGCAAAG
DYP985	DENR-P121L_SDMr	TATAGTAACCTTTTGTGGTAC
DYP1004	TMA22-FLAGf	cgatgacgacAAGTAAGCAATCCATAAATATGTAATAGCAC
DYP1005	TMA22-FLAGr	tctttgtagtcGGCAGCTCCTTCTGCAGC

Supplementary Table 5: Read preparation and alignment statistics, related to Methods.

Sample name	Description	Data type	Reads with linker (40S: 15-80 nt; 80S: 25-34 nt)	Not aligned to ncRNA	Non-duplicated reads	Aligned to genome or splice jcns
DY134Ffx	WT_rep1	40S Ribo-seq	76,534,682	10,738,363	N/A	1,070,659
DY182Ffx	WT_rep2	40S Ribo-seq	149,909,118	15,475,711	13,131,237	3,120,815
DY134Ffx182Ffx	WT_comb	40S Ribo-seq			23,869,600	4,191,474
DY135Ffx	*rpl11b*Δ_rep1	40S Ribo-seq	59,797,074	8,441,994	N/A	2,995,246
DY183Ffx	*rpl11b*Δ_rep2	40S Ribo-seq	183,216,386	15,211,788	13,058,138	3,572,847
DY135Ffx183Ffx	*rpl11b*Δ_comb	40S Ribo-seq			21,500,132	6,568,093
DY136Ffx	*tma64*Δ/*tma20*Δ_rep1	40S Ribo-seq	74,676,100	15,106,384	N/A	4,945,753
DY184Ffx	*tma64*Δ/*tma20*Δ_rep2	40S Ribo-seq	269,790,688	30,863,064	26,615,348	7,536,728
DY136Ffx184Ffx	*tma64*Δ/*tma20*Δ_comb	40S Ribo-seq			41,721,732	12,482,481
DY137Ffx	*tma64*Δ/*tma22*Δ_rep1	40S Ribo-seq	85,808,190	18,111,304	N/A	2,995,410
DY185Ffx	*tma64*Δ/*tma22*Δ_rep2	40S Ribo-seq	129,116,115	11,641,026	9,864,587	2,466,356
DY137Ffx185Ffx	*tma64*Δ/*tma22*Δ_comb	40S Ribo-seq			27,975,891	5,461,766

Sample name	Description	Data type	Reads with linker (40S: 15-80 nt; 80S: 25-34 nt)	Not aligned to ncRNA	Non-duplicated reads	Aligned to genome or splice jcns
DY154Ffx	*tma20*Δ_rep1	40S Ribo-seq	28,572,266	3,186,885	2,226,222	515,163
DY186Ffx	*tma20*Δ_rep2	40S Ribo-seq	151,412,331	14,266,655	12,220,844	3,960,529
DY154Ffx186Ffx	*tma20*Δ_comb	40S Ribo-seq			14,447,066	4,475,692
DY155Ffx	*tma22*Δ_rep1	40S Ribo-seq	76,177,581	4,903,900	3,851,596	706,983
DY187Ffx	*tma22*Δ_rep2	40S Ribo-seq	176,019,532	11,874,856	10,166,308	4,060,986
DY155Ffx187Ffx	*tma22*Δ_comb	40S Ribo-seq			14,017,904	4,767,969
DY156Ffx	*tma64*Δ_rep1	40S Ribo-seq	92,234,927	7,419,322	5,631,742	1,168,027
DY188Ffx	*tma64*Δ_rep2	40S Ribo-seq	230,989,916	19,852,139	16,355,037	3,523,015
DY156Ffx188Ffx	*tma64*Δ_comb	40S Ribo-seq			21,986,779	4,691,042
DY158Ffx	*tma22*Δ YCplac33_rep1	40S Ribo-seq	69,626,874	7,210,346	5,036,278	1,568,818
DY189Ffx	*tma22*Δ YCplac33_rep2	40S Ribo-seq	134,846,259	12,490,996	10,197,224	3,404,615
DY158Ffx189Ffx	*tma22*Δ YCplac33_comb	40S Ribo-seq			15,233,502	4,973,433

Sample name	Description	Data type	Reads with linker (40S: 15-80 nt; 80S: 25-34 nt)	Not aligned to ncRNA	Non-duplicated reads	Aligned to genome or splice jcns
DY159Ffx	*tma22*Δ sc *TMA22*_rep1	40S Ribo-seq	89,430,986	8,128,121	5,870,433	1,652,388
DY190Ffx	*tma22*Δ sc *TMA22*_rep2	40S Ribo-seq	151,197,766	11,894,692	10,252,084	2,701,834
DY159Ffx190Ffx	*tma22*Δ sc *TMA22*_comb	40S Ribo-seq			16,122,517	4,354,222
DY160Ffx	*tma22*Δ sc *tma22-C11Y*_rep1	40S Ribo-seq	54,462,747	6,275,732	4,262,570	1,297,179
DY191Ffx	*tma22*Δ sc *tma22-C11Y*_rep2	40S Ribo-seq	141,326,950	8,591,138	7,513,104	2,954,743
DY160Ffx191Ffx	*tma22*Δ sc *tma22-C11Y*_comb	40S Ribo-seq			11,775,674	4,251,922
DY161Ffx	*tma22*Δ sc *tma22-A105L*_rep1	40S Ribo-seq	76,213,283	8,653,341	5,336,711	1,722,627
DY192Ffx	*tma22*Δ sc *tma22-A105L*_rep2	40S Ribo-seq	279,189,681	19,085,447	16,066,730	3,551,850
DY161Ffx192Ffx	*tma22*Δ sc *tma22-A105L*_comb	40S Ribo-seq			21,403,441	5,274,477
DY193Ffx	*tma22*Δ sc DENR	40S Ribo-seq	122,906,664	8,589,653	7,079,797	2,268,072
DY180F	*rpl11b*Δ_rep1	80S Ribo-seq	113,036,426	38,839,746	34,487,611	26,495,860
DY181F	*rpl11b*Δ_rep2	80S Ribo-seq	64,202,799	23,644,589	20,977,590	15,967,913
DY180F181F	*rpl11b*Δ_comb	80S Ribo-seq			55,465,201	42,463,773

Sample name	Description	Data type	Reads with linker (40S: 15-80 nt; 80S: 25-34 nt)	Not aligned to ncRNA	Non-duplicated reads	Aligned to genome or splice jcns
SM008F	*tma20*Δ	80S Ribo-seq	76,526,004	43,796,476	29,475,606	23,645,712
SM009F	*tma22*Δ	80S Ribo-seq	79,732,346	39,782,152	26,091,042	20,795,426
SM010F	*tma64*Δ	80S Ribo-seq	140,125,445	77,568,781	50,101,777	40,412,103

Supplementary Table 6: Ribosome profiling datasets from previous papers, related to Figure 4 and Methods

Sample name	Description	Reference	GEO Number	Number mapped reads*
DJY0910_1	WT (2 rep's pooled)	Young et al. (2018)	GSE108942	59,966,692
68F69F	*tma64*Δ/*tma20*Δ (2 rep's pooled)	Young et al. (2018)	GSE108942	22,162,748
70F71F	*tma64*Δ/*tma22*Δ (2 rep's pooled)	Young et al. (2018)	GSE108942	17,966,108

* raw reads were reprocessed using the new bioinformatic pipeline

Supplementary Table 7: Oligonucleotides used for ribosome profiling, related to Methods.

Primer No.	Primer Name	Primer Sequence 5' - 3'
**RNA Size Marker**		
15mer	rArUrGrUrArCrArCrGrGrArGrUrCrG	
25mer	rArUrGrUrArCrArCrGrGrArGrUrCrGrArGrCrArCrCrCrGrCrA	
34mer	rArUrGrUrArCrArCrGrGrArGrUrCrGrArGrCrArCrCrCrGrCrArArCrGrCrGrArArUrG	
80mer	rArUrGrUrArCrArCrGrGrArGrUrCrGrArCrCrCrGrCrArArCrGrCrGrArUrGrUrArCrArCrGrGrArGrUrCrGrArCrCrCrGrCrArArCrGrCrGrArUrGrUrArCrArCrGrGrArGrUrCrGrArCrCrCrGrCrArArCrGrC	
**RT Primer (Original Protocol)**		
NI-NI-9	/5Phos/AGATCGGAAGAGCGTCGTGTAGGGAAAGAGTGTAGATCTCGGTGGTCGC/iSp18/CACTCA/iSp18/TTCAGACGTGTGCTCTTCCGATCTATTGATGGTGCCTACAG	
**Subtraction Oligonucleotides**		
1b	/5BioTinTEG/GGTGCACAATCGACCGATC	
2b	/5BioTinTEG/GTTTCTTTACTTATTCAATGAAGCGG	
3b	/5BioTinTEG/TATAGATGGATACGAATAAGGCGTC	
4	/5BioTinTEG/TTGTGGCGTCGCTGAACCATAG	
5	/5BioTinTEG/CAGGGGGCATGCCTGTTTGAGCGTCAT	
6	/5BioTinTEG/CGGTGCCCGAGTTGTAATTT	
**PCR Primers (Original Protocol)**		
NI-NI-2	AATGATACGGCGACCACCGAGATCTACAC	
NI-NI-13	CAAGCAGAAGACGGCATACGAGATATCGTAGTGACTGGAGTTCAGACGTGTGCTCTTCCG	
IDX8	CAAGCAGAAGACGGCATACGAGATTCAAGTGTGACTGGAGTTCAGACGTGTGCTCTTCCG	
NI-NI-11	CAAGCAGAAGACGGCATACGAGATAGCTGCGTGACTGGAGTTCAGACGTGTGCTCTTCCG	
IDX7	CAAGCAGAAGACGGCATACGAGATGATCTGGTGACTGGAGTTCAGACGTGTGCTCTTCCG	

Primer No.	Primer Name	Primer Sequence 5’ - 3’
**Linker Oligonucleotides (Modified Protocol)**		
NI-810	5´-/5Phos/NNNNNATCGTAGATCGGAAGAGCACACGTCTGAA/3ddC/	
NI-811	5´-/5Phos/NNNNNAGCTAAGATCGGAAGAGCACACGTCTGAA/3ddC/	
NI-812	5´-/5Phos/NNNNNCGTAAAGATCGGAAGAGCACACGTCTGAA/3ddC/	
NI-813	5´-/5Phos/NNNNNCTAGAAGATCGGAAGAGCACACGTCTGAA/3ddC/	
**RT Primer (Modified Protocol)**		
NI-802	5´-/5Phos/NNAGATCGGAAGAGCGTCGTGTAGGGAAAGAG/iSp18/GTGACTGGAGTTCAGACGTGTGCTC	
**PCR Primers (Modified Protocol)**		
NI-NI-798	5´- AATGATACGGCGACCACCGAGATCTACACTCTTTCCCTACACGACGCTC	
NI-799	5´-CAAGCAGAAGACGGCATACGAGATCGTGATGTGACTGGAGTTCAGACGTGTG	
