# Supplementary material for: 40S ribosome profiling reveals distinct roles for Tma20/Tma22 (MCT-1/DENR) and Tma64 (eIF2D) in 40S subunit recycling
Source: Nat Commun. 2021 May 20;12:2976. doi: 10.1038/s41467-021-23223-8 (PMC8137927; doi:10.1038/s41467-021-23223-8)
Supplement: Supplementary file 2 — Description of Additional Supplementary Files [file 41467_2021_23223_MOESM2_ESM.pdf]

Description of Additional Supplementary Files

File Name: Supplementary Data 1
Description: Average pause ratios by penultimate codon identity
