# Supplementary material for: 40S ribosome profiling reveals distinct roles for Tma20/Tma22 (MCT-1/DENR) and Tma64 (eIF2D) in 40S subunit recycling
Source: Nat Commun. 2021 May 20;12:2976. doi: 10.1038/s41467-021-23223-8 (PMC8137927; doi:10.1038/s41467-021-23223-8)
Supplement: Supplementary file 4 — Reporting Summary [file 41467_2021_23223_MOESM4_ESM.pdf]

nature research

Corresponding author(s): Nicholas R. Guydosh

Last updated by author(s): Mar 2, 2021

Reporting Summary

Nature Research wishes to improve the reproducibility of the work that we publish. This form provides structure for consistency and transparency in reporting. For further information on Nature Research policies, see our Editorial Policies and the Editorial Policy Checklist.

Statistics

For all statistical analyses, confirm that the following items are present in the figure legend, table legend, main text, or Methods section.

n/a	Confirmed	
☐	☒	The exact sample size (*n*) for each experimental group/condition, given as a discrete number and unit of measurement
☐	☒	A statement on whether measurements were taken from distinct samples or whether the same sample was measured repeatedly
☐	☒	The statistical test(s) used AND whether they are one- or two-sided<br>*Only common tests should be described solely by name; describe more complex techniques in the Methods section.*
☐	☒	A description of all covariates tested
☐	☒	A description of any assumptions or corrections, such as tests of normality and adjustment for multiple comparisons
☐	☒	A full description of the statistical parameters including central tendency (e.g. means) or other basic estimates (e.g. regression coefficient) AND variation (e.g. standard deviation) or associated estimates of uncertainty (e.g. confidence intervals)
☐	☒	For null hypothesis testing, the test statistic (e.g. *F*, *t*, *r*) with confidence intervals, effect sizes, degrees of freedom and *P* value noted<br>*Give P values as exact values whenever suitable.*
☒	☐	For Bayesian analysis, information on the choice of priors and Markov chain Monte Carlo settings
☒	☐	For hierarchical and complex designs, identification of the appropriate level for tests and full reporting of outcomes
☐	☒	Estimates of effect sizes (e.g. Cohen's *d*, Pearson's *r*), indicating how they were calculated

Our web collection on statistics for biologists contains articles on many of the points above.

Software and code

Policy information about availability of computer code

Data collection	Sequencing data was collected on Illumina HiSeq 2500 and 3000 machines by the NIDDK Genomics Core and the NHLBI DNA Sequencing and Genomics Core.
Data analysis	Biopython Version 1.72<br>CutAdapt Version 1.18<br>Bowtie Version 1.1.2 (Langmead et al. 2009)<br>Custom code is available on Github: https://github.com/guydoshlab<br>Igor Pro Version 8.04 (WaveMetrics)

For manuscripts utilizing custom algorithms or software that are central to the research but not yet described in published literature, software must be made available to editors and reviewers. We strongly encourage code deposition in a community repository (e.g. GitHub). See the Nature Research guidelines for submitting code & software for further information.

Data

Policy information about availability of data

All manuscripts must include a data availability statement. This statement should provide the following information, where applicable:

- Accession codes, unique identifiers, or web links for publicly available datasets
- A list of figures that have associated raw data
- A description of any restrictions on data availability

Raw and analyzed data have been deposited in the NCBI GEO database under the accession number GSE145904 (https://www.ncbi.nlm.nih.gov/geo/query/acc.cgi?acc=GSE145904). Raw and analyzed data for ribosome profiling datasets from previous papers (see Table S6) are available from the NCBI GEO database under the

accession number GSE108942 (https://www.ncbi.nlm.nih.gov/geo/query/acc.cgi?acc=GSE108942).

Field-specific reporting

Please select the one below that is the best fit for your research. If you are not sure, read the appropriate sections before making your selection.

☒ Life sciences ☐ Behavioural & social sciences ☐ Ecological, evolutionary & environmental sciences

For a reference copy of the document with all sections, see nature.com/documents/nr-reporting-summary-flat.pdf

Life sciences study design

All studies must disclose on these points even when the disclosure is negative.

Sample size	40S ribosome profiling was performed in duplicate (2 biological replicates)
Data exclusions	When available, replicate datasets were combined to enhance sequencing depth. However, 2-D metagene plots (Figures 1c and 3c), some GCN4 analysis (Figures S3a top and S3b), and examples of N-terminal extension (Figures 2b and S2) relied on the rep_2 datasets only since individual peaks were sharper and aided analysis. Thresholding was used in some cases to eliminate artefacts, as described in methods.
Replication	40S ribosome profiling was performed in duplicate. All westerns were repeated at least twice on two independently grown cultures for each condition. All 40S ribosome profiling and western replications were successful.
Randomization	Randomization is not relevant. We did not deal with human or animal subjects and analysis was automated.
Blinding	Blinding is not relevant. We did not deal with human or animal subjects and analysis was automated.

Reporting for specific materials, systems and methods

We require information from authors about some types of materials, experimental systems and methods used in many studies. Here, indicate whether each material, system or method listed is relevant to your study. If you are not sure if a list item applies to your research, read the appropriate section before selecting a response.

Materials & experimental systems

n/a	Involved in the study	
☐	☒	Antibodies
☒	☐	Eukaryotic cell lines
☒	☐	Palaeontology and archaeology
☒	☐	Animals and other organisms
☒	☐	Human research participants
☒	☐	Clinical data
☒	☐	Dual use research of concern

Methods

n/a	Involved in the study	
☒	☐	ChIP-seq
☒	☐	Flow cytometry
☒	☐	MRI-based neuroimaging

Antibodies

Antibodies used	Mouse monoclonal antibody Anti-FLAG (Sigma; F1804)<br>Mouse monoclonal antibody Anti-VDAC1/Porin (Abcam; ab110326)<br>Mouse monoclonal antibody Anti-DENR (Abnova; H00008562-M01)<br>Mouse monoclonal antibody Anti-beta actin (Abcam; ab8224)
Validation	Affinity Purified Anti-FLAG antibody (Product Code F1804), Western blot immunostaining was utilized. Purified FLAG-BAP, an N-terminal FLAG fusion protein of E. coli bacterial alkaline phosphatase (49.3 kDa), was spiked into mammalian CHO lysate. SDS-electrophoresis was performed and the separated proteins transferred to a nitrocellulose membrane. The new ANTI-FLAG M2 was used to probe for the FLAG-BAP fusion protein, followed by a rabbit anti-mouse HRP conjugate (Product Code A9044).<br><br>Anti-VDAC1/Porin - Immunogen: Full length native protein (purified). This information is considered to be commercially sensitive.<br>Anti-VDAC1/Porin antibody [16G9E6BC4] - Mitochondrial Loading Control (ab110326) at 1 µg/ml + Yeast spheroplast membrane fraction. Predicted band size: 30 kDa<br><br>DENR (NP_003668, 1 a.a. ~ 81 a.a) partial recombinant protein with GST tag. MW of the GST tag alone is 26 KDa. Antibody Reactive Against Recombinant Protein. Western Blot detection against Immunogen (34.65 KDa).<br><br>Synthetic peptide corresponding to Human beta Actin aa 1-100 (N terminal) conjugated to keyhole limpet haemocyanin (Sulfosuccinimidyl 4-N-maleimidomethyl-cyclohexane-1-carboxylate (Sulfo-SMCC)). WB: A431; HEK293; NIH3T3; PC12 whole cell

lysates; Xenopus embryo lysate; Drosophila lysate; S. pombe lysate. Flow Cyt: HeLa cells. ICC/IF: Panc-1 cells; Human fibroblasts. IHC/P: Human colon (FFPE). Recognises a single band at 42kD representing beta Actin.
